# Mesenchymal Stromal Cells Primed with Paclitaxel Provide a New Approach for Cancer Therapy

**DOI:** 10.1371/journal.pone.0028321

**Published:** 2011-12-20

**Authors:** Augusto Pessina, Arianna Bonomi, Valentina Coccè, Gloria Invernici, Stefania Navone, Loredana Cavicchini, Francesca Sisto, Maura Ferrari, Lucia Viganò, Alberta Locatelli, Emilio Ciusani, Graziella Cappelletti, Daniele Cartelli, Caruso Arnaldo, Eugenio Parati, Giovanni Marfia, Roberto Pallini, Maria Laura Falchetti, Giulio Alessandri

**Affiliations:** 1 Department of Public Health, Microbiology, Virology, University of Milan, Milan, Italy; 2 Department of Cerebrovascular Diseases, Fondazione IRCCS Neurological Institute Carlo Besta, Milan, Italy; 3 Istituto Zooprofilattico Sperimentale della Lombardia e dell' Emilia Romagna, Brescia, Italy; 4 Fondazione IRCCS Istituto Nazionale dei Tumori, Milan, Italy; 5 Department of Diagnostics and Applied Technology, Fondazione IRCCS Neurological Institute Carlo Besta, Milan, Italy; 6 Department of Biology, University of Milan, Milan, Italy; 7 Department of Experimental and Applied Medicine, University of Brescia, Brescia, Italy; 8 Institute of Neurosurgery, Catholic University School of Medicine, Rome, Italy; 9 Institute of Neurobiology and Molecular Medicine, CNR, Rome, Italy; University of Frankfurt - University Hospital Frankfurt, Germany

## Abstract

**Background:**

Mesenchymal stromal cells may represent an ideal candidate to deliver anti-cancer drugs. In a previous study, we demonstrated that exposure of mouse bone marrow derived stromal cells to Doxorubicin led them to acquire anti-proliferative potential towards co-cultured haematopoietic stem cells (HSCs). We thus hypothesized whether freshly isolated human bone marrow Mesenchymal stem cells (hMSCs) and mature murine stromal cells (SR4987 line) primed *in vitro* with anti-cancer drugs and then localized near cancer cells, could inhibit proliferation.

**Methods and Principal Findings:**

Paclitaxel (PTX) was used to prime culture of hMSCs and SR4987. Incorporation of PTX into hMSCs was studied by using FICT-labelled-PTX and analyzed by FACS and confocal microscopy. Release of PTX in culture medium by PTX primed hMSCs (hMSCsPTX) was investigated by HPLC. Culture of Endothelial cells (ECs) and aorta ring assay were used to test the anti-angiogenic activity of hMSCsPTX and PTX primed SR4987(SR4987PTX), while anti-tumor activity was tested *in vitro* on the proliferation of different tumor cell lines and in vivo by co-transplanting hMSCsPTX and SR4987PTX with cancer cells in mice. Nevertheless, despite a loss of cells due to chemo-induced apoptosis, both hMSCs and SR4987 were able to rapidly incorporate PTX and could slowly release PTX in the culture medium in a time dependent manner. PTX primed cells acquired a potent anti-tumor and anti-angiogenic activity *in vitro* that was dose dependent, and demonstrable by using their conditioned medium or by co-culture assay. Finally, hMSCsPTX and SR4987PTX co-injected with human cancer cells (DU145 and U87MG) and mouse melanoma cells (B16) in immunodeficient and in syngenic mice significantly delayed tumor takes and reduced tumor growth.

**Conclusions:**

These data demonstrate, for the first time, that without any genetic manipulation, mesenchymal stromal cells can uptake and subsequently slowly release PTX. This may lead to potential new tools to increase efficacy of cancer therapy.

## Introduction

The main goal in cancer chemotherapy is to localize the drug effect in the tumor microenvironment in order to kill as many cancer cells as possible while producing the lowest collateral toxicity. To do this, a significant number of technical approaches, from the use of toxic immunoconjugates for targeting tumor specific antigens [1] to the sophisticated use of nanoparticles [2] or manipulated stem cells [3] for drugs delivery, have been investigated and published in the last 20 years. Since Mesenchymal stem cells (MSCs) easily adapt to culture conditions necessary for *in vitro* manipulation and home to pathological tissues when injected *in vivo*, these cells seem to represent the best choice to deliver anti-tumor agents [4], [5]. Transgenic procedures have been used to induce MSCs to secrete therapeutic cytokines or growth and inhibitory factors [6], [7]. Recent data have shown that engineered MSCs produce anti-tumor factors which have the capacity to kill cancer cells both *in vitro* and *in vivo* [3], [8]–[12]. Although there are promising results on animals, the genetic manipulation of MSCs in clinical application in humans has some risks [13].

In a previous study, we demonstrated that mouse bone marrow (BM) derived stromal cell (SR4987 cell line) cultured in the presence of doxorubicin (DXR), a potent anti-cancer compound, were able to uptake significant amounts of the drug without showing significant signs of toxicity. In contrast, hematopoietic stem cells (HSCs) from BM were very sensitive to DXR. Interestingly, we observed a significant inhibition of HSCs-induced colony formation units (CFU) if co-cultured with SR4987 first primed with DXR. We concluded that murine stromal cells may act as a reservoir for DXR that, subsequently, may release some DXR metabolites or even DXR in its original form, leading to HSCs-induced CFU inhibition [14]. Consequently, we hypothesized that, also *in vivo*, BM stromal cells may play a dual role in controlling drug toxicity, depending on their capacity to uptake and release chemotherapeutic drugs [14]. Considering these properties, we here investigate whether human MSCs (hMSCs) freshly prepared from BM and mouse SR4987, after priming with the anticancer and anti-angiogenic drug paclitaxel (PTX), may acquire the capacity to kill tumor cells (TCs) in their proximity.

Paclitaxel is a highly lipophilic drug (derived from *Taxus brevifolia*), very active on many solid tumors and also able to inhibit endothelial cell proliferation. Paclitaxel affects cytoskeleton by promoting microtubule polymerization that induces the mitotic arrest of the cell [15], [16].

We demonstrate, for the first time, that, in a time dependent kinetics, hMSCs and mouse SR4987 primed with PTX (MSCsPTX, SR4987PTX) release the drug in an amount enough to affect tumor proliferation, to kill endothelial cells (ECs) *in vitro* and, most importantly, to reduce tumor growth *in vivo*. Our results are the first demonstration that, through a simple *in vitro* procedure, hMSCs and mouse stromal cells can be loaded with anti-cancer drugs and used *in vivo* to release them into a tumor microenvironment.

## Results

### Characterization of MSCs, P-glycoprotein (P-gp) expression and sensitivity to PTX

**Figure 1:**
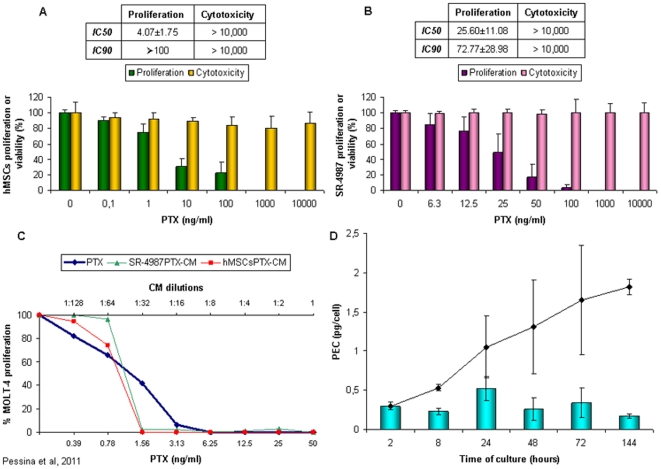
PTX priming of hMSCs and SR4987 does not affect viability and induces anti-proliferative activity on MOLT-4. Different concentrations of PTX (from 0.1 to 10.000 ng/ml) were added to culture of hMSCs (Fig. 1A) and mouse SR4987 (Fig. 1B). The cytotoxic activity was evaluated at 24 h of treatment; the effect on proliferation at 7 day of culture by a MTT assay. Optical Density measured in cultures of MSC that did not receive PTX were considered as 100% proliferation. Note that concentration of PTX up to 10.000 ng/ml did not affect either hMSCs or SR4987 cell viability. The small tables insert in the A and B indicate the IC_50_ and IC_90_ induced by PTX on hMSCs and SR4987 respectively. Both the conditioned media (CM) from PTX primed cells (hMSCsPTX-CM and SR4987PTX-CM) produced a dose dependent growth inhibition of MOLT-4 reported (Fig. 1C) as percent of that produced by CM from untreated cells (hMSCs-CM and SR4987-CM). Note that at 1∶16 dilution of both hMSCsPTX-CM and SR4987PTX-CM produced 100% growth inhibition equal to those obtained with 3.13 ng/ml of PTX. This biological assay was used to estimate PEC released by single PTX treated hMSC and its accumulation in the culture medium during the time (D). The histogram indicates PEC released by hMSCPTX at different times of culture. The curve indicates the PEC accumulation in the hMSCsPTX-CM. Note that each hMSCPTX releases around 1 pg of PEC in 24 h, reaching a maximal accumulation of around 1.7 pg after 144 h. Value are the mean ± standard deviation (SD) of five independent experiments.

The cells used in this study were three different fresh preparations of hMSCs and the mouse stromal cell line SR4987 [17]. Cultured hMSCs were analyzed to confirm the expression of MSC markers as well as their multipotential differentiation capacity. They were positive for CD44+, CD73+, CD90+, CD105+, and HLA-I and negative for CD14−, CD31−, CD34−, CD45−, CD80− and HLA-II. When cultured under differentiating conditions, they acquired osteo-adipo and chondroblasts markers (Fig. S1). We next assessed the sensitivity of hMSCs and SR4987 to PTX, in a 24 h cytotoxicity test and in an anti-proliferation assay at 7 days (Fig. 1A, B). SR4987 and hMSCs were sensitive to the anti-proliferative activity of PTX, according to a dose-dependent kinetics with a IC_50_ value of 25.6±11.08 ng/ml and 4.07±1.75 ng/ml respectively. By contrast, both SR4987 and hMSCs were strongly resistant to PTX direct cytotoxicity, with very little cell death even at concentrations higher than 10.000 ng/ml (Fig. 1A, B). The inhibition of hMSCs and SR4987 proliferation by PTX was confirmed by performing cell cycle analysis, showing significant accumulation of cells in S, and in a minor degree, G2/M phase. The viability of both hMSCs and SR4987 cells was not affected significantly and cells detached, washed and subcultured in the absence of drug gave a cell monolayer with a cell viability in the range of controls and a cell cycle pattern restored after 72 h (Fig. S2). These data have been confirmed by the percentages of apoptotic/necrotic cells counted in the different experimental conditions by Annexin assay (Table S1). The treatment does produce some loss of cells due to chemo-induced apoptosis, although the only significant increase in apoptosis (P<0.05) was evidenced in SR4987 cells (at 24 hours of treatment with PTX) that recovered after drug replacement (24+24 h). These data are in agreement with the reports of other authors on the sensitivity of stromal cells to Paclitaxel [18], [19].

Both hMSCs and SR4987 cells expressed P-gp that was down-modulated by treatment with PTX and with verapamil (VP). The presence of VP increased SR4987 sensitivity to the anti-proliferation activity of PTX, whereas it was not effective on hMSCs (Fig. S3).

### PTX-uptake and release by hMSCs

Based on previous studies [14], sub-confluent culture (3×10^5^) of adherent hMSCs were exposed for 24 h to 2.000 ng/ml PTX (enough to completely block cell proliferation, but not able to affect cell viability). After several washes and trypsinization, hMSCsPTX were further cultured for 24 h and their conditioned medium (CM) was tested on Molt-4, a leukaemia cell line very sensitive to PTX (IC_50_ of 1.48±1.06 ng/ml) [20]. The CM from both cultures of hMSCPTX (hMSCsPTX-CM) produced a strong dose-dependent anti-proliferative effect on MOLT-4, equivalent to those obtained with pure PTX at doses from 0.39 to 50 ng/ml (Fig. 1C). By contrast, the CM from control cells (hMSCs-CM) were not effective. Comparing the inhibitory activity of pure PTX and CM on Molt-4, we calculated the PTX equivalent concentration (PEC) in the CM used to estimate the PEC released by a single cell (PEC pg/cell). The total PEC internalized by hMSCsPTX in 24 h, evaluated by testing the hMSCsPTX cell lysates, was 2.67±0.8 pg/cell, suggesting that hMSCs in 24 h could incorporate about 8% of the PTX initially available (33.3 pg/cell). Results on lysates of hMSCs fixed in formalin before PTX priming indicated some unspecific binding of PTX, corresponding to 0.11±0.01 of PEC pg/cell (about 4% of total PEC present in the lysate of living cells).

**Figure 2:**
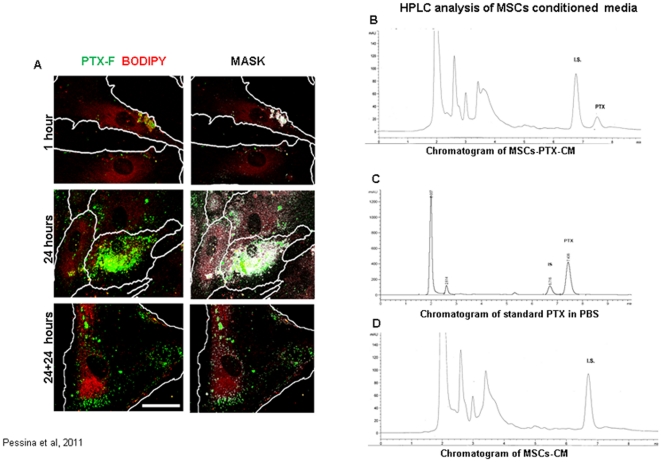
PTX-F internalization and release by hMSCs. The internalization of PTX-F was analyzed by confocal microscopy (A) in live hMSCs primed 1 (1) or 24 (24) h with PTX-F (green) and loaded with the Golgi specific marker BOPIPY®TR ceramide (red). Cells were also observed 24 h after washing step (24+24). PTX-F accumulates in cells and co-localizes with Golgi apparatus or Golgi-derived vesicles. Mask panel highlights the co-localization between PTX-F and BODIPY®TR ceramide showing white spots, that indicate those pixels in which both the fluorescent signals are detectable.. White lines represent the cell boundary and arrows indicate vesicles close to the cell membrane. Scale bar: 20 µm. The release of PTX in the hMSCsPTX-CM at 24 h was analysed by HPLC. The elution profile (B) was compared to that of pure PTX at 1.000 ng/ml (C). The figure reports a chromatogram profile of one typical experiment where hMSCsPTX-CM evidences a peak that clearly identified PTX and that was quantified on a PTX standard curve as 68.1 ng/ml. Figure 2D reports the profile of the hMSCs-CM cultured in the absence of PTX. The peak labeled as I.S. is the internal standard Cephalomannine added to all samples for the correct quantification of PTX.

Thereafter, we calculated the time dependent release of PEC by hMSCsPTX by replacing and collecting their CM at different intervals of time. Detectable activity of PEC was present after 2 h of incubation of hMSCsPTX reaching a PEC of 1 pg/cell during the first 24 h of culture, and a maximum concentration of about 1.7–2.0 pg/cell at 144 h (Fig. 1D). Since these values did not increase with longer incubation, we estimated that around 25–30% of the total PEC found in cell lysate was retained by the cells and never released. The internalization of PTX into hMSCs was investigated by confocal microscopy using Fluorescent PTX (PTX-F) (Fig. 2A). PTX-F localization into hMSCs was analysed over time. After 1 h of priming, the internalization of PTX-F by hMSCs was appreciable. The staining was intense and enriched inside vesicles at the end of priming (24 h). After 24 h, we observed that the distribution of PTX-F remained confined to vesicles, many of which were close to the cell membrane, suggesting a possible secretion. To assess if PTX-F was enriched in vesicles derived from Golgi apparatus, co-localization analysis was performed in hMSCs stained with the specific marker BODIPY® TR ceramide. As seen in the white spots in Figure 2A mask, we observed a high level of colocalization between PTX-F and BODIPY® TR ceramide, meaning that PTX-F was internalized inside Golgi-derived vesicles. Thus, PTX-F accumulates in vesicles instead of accumulating in microtubules [15] as many other xenobiotics do [21], and its levels decrease in hMSCs following the kinetics shown by FACS analysis (Fig. 2A and Fig. S4).

The presence of PTX in hMSCsPTX-CM was confirmed by HPLC analysis. The HPLC chromatograms obtained from hMSCsPTX-CM and from a standard sample of PTX in PBS (1.000 ng/ml) (Fig. 2B, C) show that a peak of identical retention time of PTX was eluted from the hMSCsPTX-CM. HPLC analysis revealed the presence of other nonspecific peaks (2.5–4 minutes) probably due to compounds produced by cells and not correlated to the presence of PTX because also present in the chromatogram of control medium hMSCs-CM (Fig. 2D). The presence of the main PTX metabolite, the 6 alpha-hydroxy-paclitaxel, normally eluted at 5.5 minutes and of others PTX metabolites can be excluded [22].

A similar PTX priming technique was applied to the mouse SR4987 that showed a similar kinetics of incorporation and release of PTX with a higher trend of drug release during the first 24 h of incubation (data not shown).

### HMSCsPTX and SR4987PTX inhibit proliferation of different kinds of TCs *in vitro*

**Figure 3:**
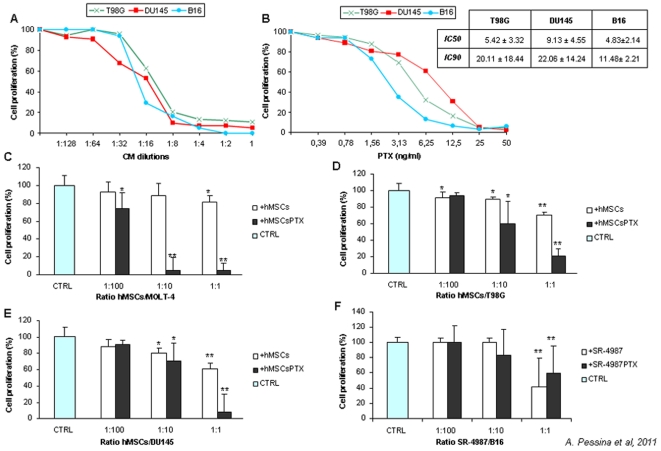
Primed hMSCsPTX and SR4987PTX inhibit proliferation of different TC lines in vitro. In (A) is shown the kinetics of growth inhibition induced by serial dilutions of hMSCsPTX-CM on T98G, DU145 and of SR4987PTX-CM on B16 which was compared with activity of different concentrations of PTX on the same TCs (B). The CM addition produced a strong anti-proliferative effect on all TCs tested in a dose dependent manner: 1∶16 and 1∶4 dilutions IC_50_ and IC_90_ growth inhibition on all TC lines respectively, corresponding to the PTX concentrations necessary to obtain IC_50_ and IC_90_ on the different tumor cells as reported in small table insert in (B). Inhibition of TCs proliferation was also obtained by a direct co-culture assay. Primed hMSCsPTX mixed, at different ratios (1∶100–1∶10–1∶1 MSCs/TCs), with MOLT-4 (C), T98G (D), DU145 (E) showed dose dependent capacity to block TCs proliferation evaluated in a MTT test at 7 days expressed as percent of OD measured for TCs cultured in control medium alone (CTR) or in presence of not primed hMSCs. SR4987PTX behaved like hMSCsPTX. However, even not primed SR4987 per se showed some anti-proliferative capacity on B16 melanoma at 1∶1 ratio (F). The histograms report the mean ± SD of three experiments with the statistical significance as follows: * ( p<0.05),** ( p<0.01) vs tumor cell growth (CTRL).

To evaluate the *in vitro* anti-tumor potential of hMSCsPTX and mouse SR4987PTX, we then investigated the effect of hMSCsPTX-CM on two human cell lines (DU145, T98G) and of SR4987PTX-CM on mouse B16 melanoma cell line. As shown in Fig. 3, the addition of hMSCsPTX-CM at 1∶4 to 1∶8 dilution induced up to 80% of growth inhibition on all tumor cell lines (Fig. 3A). At 1∶2 dilution, equivalent to the addition of about 25 ng/ml of pure PTX, 100% of tumor growth inhibition was obtained (Fig. 3B). Combining these data with those from the kinetics release (Fig. 1D), we could estimate that, in 24 hrs, 3×10^5^ hMSCsPTX may release around 50–60 ng/ml of PEC which are concentrations 3–5 fold the IC_90_ values of PTX for the cancer cells studied. The addition of control hMSCs-CM did not affect TCs proliferation (data not shown). The capacity of hMSCsPTX to inhibit different TCs proliferation was confirmed by co-culture assay in which hMSCsPTX and TCs were mixed at different ratios (from 1∶1 to 1∶100). The presence of hMSCsPTX inhibited proliferation of all tumor cell lines tested, according to their proportional presence (Fig. 3C, D, E). This allowed us to calculate an arbitrary value expressed as a ratio between hMSCsPTX and TCs that produced IC_50_. The IC_50_ values were 1∶47 for MOLT-4, 1∶5 for T98G and DU145 and only 1∶2–3 for B16 tumor cells. The addition of control hMSCs did not substantially affect TCs proliferation, although a low significant growth inhibition (p<0.02) was observed at 1∶1 ratio for all the tumor cell lines tested.

Mouse SR4987PTX worked like hMSCsPTX in uptake and release of the drug as tested by MTT assay on CM (Fig. 1A). However, in the co-culture assay, SR4987 cells “per se” produced a inhibition of B16 melanoma cells ( p<0.05) that did not differ from that produced by SR4987PTX (Fig. 3F).

### hMSCSPTX and mouse SR4987PTX display potent *in vitro* anti-angiogenic activity

**Figure 4:**
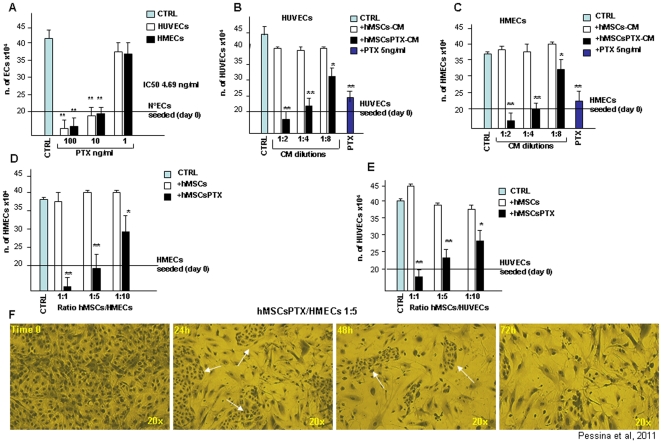
PTX and primed hMSCsPTX inhibit ECs proliferation in vitro. PTX inhibits ECs proliferation (A). HUVECs and HMECs (2×10^5^) were cultured for 72 h in the presence of different concentration of PTX. The IC_50_ was around 4.69 ng/ml of PTX and at 10 ng/ml PTX significantly blocked both HUVECs and HMECs proliferation, which at higher doses was cytotoxic. Similarly, the addition of hMSCsPTX-CM greatly inhibit HUVECs (B) and HMECs (C) proliferation. At 1∶2 dilution, hMSCsPTX-CM was cytotoxic, while at higher 1∶4 and 1∶8 dilutions significantly inhibit ECs proliferation. Inhibition of ECs proliferation was obtained by co-culture assay. HMSCsPTX co-cultured at 1∶1 and 1∶5 ratio (MSCs/ECs) was cytotoxic for both HUVECs (D) and HMECs (E). At 1∶10 ratio hMSCsPTX continued to inhibit ECs proliferation. Untreated control hMSCs did not affect ECs. In (F) photographs of culture of hMSCsPTX mixed 1∶5 with HMECs. Cells fixed and stained at different time intervals are shown. hMSCsPTX kill HMECs as early as 24 h after seeding. White arrows indicate the islands of HMECs still present in the culture. Note that after 72 h most of the HMECs seeded were killed and only hMSCsPTX remain in the culture (20× magnification). Bars in the figures are the means ± SD of three separate experiments done in triplicate. * p<0.05, **p<0.01 vs untreated hMSCs.

Because PTX is considered an inhibitor of angiogenesis [16], [23], we thus investigated whether hMSCsPTX and SR4987PTX could affect angiogenesis *in vitro* (Fig. 4). The anti-angiogenic activity of pure PTX was initially tested on HUVECs and on microvascular ECs (HMECs) proliferation. PTX at doses up to 10 ng/ml was extremely cytotoxic for both HUVECs and HMECs. PTX produced around 50% of ECs growth inhibition (IC_50_) at 4.6 ng/ml (Fig. 4A). The effect of hMSCsPTX-CM on HUVECs and HMECs was extremely cytotoxic at 1∶2 and 1∶4 dilutions (Fig. 4B, C). At 1∶8 hMSCsPTX-CM inhibited both HUVECs and HMECs proliferation but less than 5 ng/ml of pure PTX. Similar results were obtained by co-culture assay (Fig. 4D, E, F). Ratio 1∶1 and 1∶5 of hMSCsPTX/ECs produced a strong cytotoxic effect on both HUVECs and HMECs, while at ratio 1∶10, no cytotoxicity but significant ECs growth inhibition was observed (Fig. 4D, E). Interestingly, at ratio 1∶5, hMSCsPTX initiated killing HMECs during the first 24 h of co-culture; after 72 h incubation very few HMECs survived (Fig. 4F). Control hMSCs-CM did not affect ECs proliferation.

**Figure 5:**
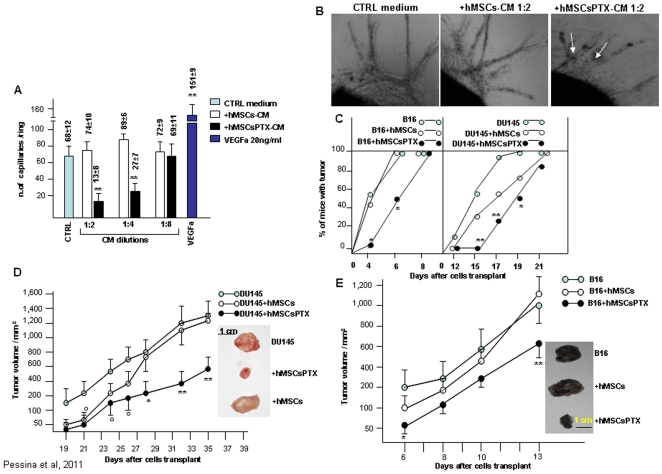
HMSCsPTX inhibit microvessel out-growth in vitro and reduce tumor growth in vivo. In (A) and (B) rat aorta ring assay was used to test hMSCsPTX-CM microvessel growth inhibition. In (A) hMSCsPTX-CM at different dilutions added to rat aorta rings in the presence or in the absence of VEGFa induced a great reduction of capillary outgrowth compared to CTRL medium and to hMSCs-CM. VEGFa 20 ng/ml was used as positive controls (**p<0.01 vs hMSCs-CM). In (B) photographs show hMSCsPTX-CM (at 1∶2 dilution), which induced capillary regression as demonstrated by the presence of vessel rupture and necrotic zones (arrows) (magnifications 20×). In (C), (D) and (E) the effects of hMSCsPTX *in vivo* on tumor takes (C), on DU145 (D) and B16 (E) growth are shown. Around 0.4×10^6^ hMSCsPTX and control untreated hMSCs were mixed (at ratio 1∶5 hMSCs/TCs/) with 2×10^6^ DU145 or B16 and then injected subcutaneously (s.c.) into mice. Tumor volumes were calculated by measuring the tumor diameters taken every two days with a calibre. The co-injection of hMSCsPTX with DU145 or B16, produced a significant delay of tumor appearance (C) as well as a great reduction of DU145 (D) and B16 (E) tumor volume, whereas the co-injection with untreated hMSCs did not affect either DU145 or B16 volumes, nor did the injection of TCs mixed 5 minutes before injection with 2.000 ng/ml of PTX (see Table 1). The insert in (D) and (E) is the photo of tumors of control, hMSCsPTX and hMSCs treated mice at time of sacrifice. *p<0.05 and **p<0.01 vs TCs alone or *vs* hMSCs treated mice.

The anti-angiogenic potential of hMSCsPTX was also investigated by using the rat aorta ring assay [24]. As shown in Fig. 5A, the addition of 1∶2 and 1∶4 dilution of hMSCsPTX-CM strongly reduced either spontaneous or VEGFa-induced sprouting of neocapillaries from aorta rings. Dilution 1∶8 of hMSCsPTX-CM that inhibit ECs proliferation did not affect aorta ring capillaries formation (Fig. 5A). HMSCsPTX-CM was also able to induce capillary regression if added to aorta rings after 7 days of culture. Under microscopic examination, several zones of vessel necrosis were observed (Fig. 5B). The addition of hMSCs-CM did not substantially affect capillary formation (Fig. 5A). Similar results with SR4987PTX were obtained (Fig. S5).

### hMSCsPTX and mouse SR4987PTX inhibit tumor growth *in vivo*

*In vitro* data demonstrated that both hMSCsPTX and SR4987PTX were strongly inhibitory for TCs and even cytotoxic for ECs. To see whether they may affect tumor growth *in vivo*, hMSCsPTX and control hMSCs were co-injected at ratio 1∶5 either with human DU145 or mouse B16 melanoma cancer cells in immunodeficient mice. Control groups were also injected with TCs alone or TCs mixed, 1–2 minutes before injection, with 2.000 ng/ml of free PTX. In Table 1 and in Figure 5 the results are summarized. HMSCsPTX mixed either with DU145 or B16 melanoma produced a significant delay in tumor takes (Fig. 5C) and reduced both DU145 (Fig. 5D) and B16 tumor growth (Fig. 5E). The anti-tumor effect on DU145 induced by hMSCsPTX was more potent than that on B16. However, tumor weights of DU145 and B16 were significantly reduced by hMSCsPTX treatment; 25% of mice treated with DU145 and hMSCsPTX were also tumor free (Table 1). Interestingly, the co-injection of DU145 and B16 cells with a high concentration of free PTX did not delay tumor takes or affect growth (Table 1) which suggests that time-dependent release of PTX by hMSCsPTX is necessary to affect cancer cells proliferation. This may also occur *in vivo*.

**Table 1:** Anti-tumor activity of hMSCs PTX and SR4987PTX.

Groups MSCs/origin	Tumor model	Animal Strain	Ratio MSCs/TCs	TW (gr±sd)	TT (%)	Number of mice/group
Control	DU145	Nod Scid	-	0.998±0.276	100	8
+PTX 2000 ng/ml			-	1.243±0.311	100	5
+hMSCs			1∶5	1.043±0.231	100	6
+hMSCsPTX			1∶5	**0.468±0.102** **	75^(°)^	8
Control	B16	Nude Mice	-	1.016±0.230	100	10
+PTX 2000 ng/ml			-	1.105±0.212	100	6
+hMSCs			1∶5	1.118±0.356	100	10
+hMSCsPTX			1∶5	**0.657±0.245** *	100	9
Control	B16	C57BL/6	-	2.220±0.890	100	8
+PTX 2000 ng/ml			-	1.965±0.547	100	5
+SR4987			1∶5	**0.830±0.310** *	100	6
+SR4987PTX			1∶5	**0.450±0.310** **	100	6

HMSCs and mouse SR4987 were used to study anti-tumor activity. C57BL/6 and Nude mice received s.c. B16 melanoma cells at concentration of 2×10^5^ and 2×10^6^/0.2 ml saline respectively. Nod/Scid mice were injected s.c. with a human prostate carcinoma cells DU145 at the concentration of 2×10^6^/0.2 ml saline. HMSCs and SR4987 primed or not with PTX were mixed with TCs 5 minutes before injection at the ratio (MSCs/TCs) shown in the Table. Similarly TCs were also mixed with PTX free at 2.000 ng/ml 5 minutes before injection. Nude mice injected with hMSCs and B16 melanoma cells were sacrificed on day 14 and Nod/Scid mice treated with human DU145 and hMSCs were sacrificed 40 days after cells injection. C57BL/6 mice receiving syngenic SR4987 and B16 melanoma cells were sacrificed on day 32. For each group of mice, tumors were excised and weighed. Numbers in the table are the means of tumor weight (TW) ± standard deviation (SD). Tumor takes (TT) indicate the % of mice with tumor at the time of sacrifice.

(°): two mice were tumor free at time of sacrifice.

*p<0.05.

**p<0.01 v.s. Controls (TCs treated without MSCs).

In C57BL/6 model SR4987PTX vs SR4987 was not significant.

Similar results were obtained by using mouse SR4987 on B16 melanoma (Table 1). SR4987PTX co-injected with B16 (at ratio 1∶5) into C57B16 mice, which are syngenic for SR4987 [14], significantly delayed tumour takes and reduced B16 growth, although the co-injection with control SR4987 “per se” produced some inhibitory activity on B16 growth (Fig. S6).

### Effects of mouse SR4987PTX on subcutaneous xenografts of glioblastoma U87MG cells

Experiments using RFP+ U87MG glioblastoma cells and GFP+ SR4987 were designed in order to trace the two cell fractions and to investigate their interactions *in vivo*. Control mice, grafted either with U87MG cells or with U87MG cells and SR4987, demonstrated that the expression of RFP and GFP did not alter tumorigenicity and survival of these cells in the *in vivo* condition. Overall, the SR4987PTX cells had an inhibitory effect on the growth of glioblastoma xenografts (Fig. S7A–B). At the 2, 4, and 6-week time points, the RFP+ U87MG/GFP+ SR4987PTX xenografts were significantly smaller than the RFP+ U87MG xenografts (*p*<0.01, *p*<0.05, and *p*<0.001, respectively; Student's *t*-test). At the same time points, the RFP+ U87MG/GFP+ SR4987-PTX xenografts were significantly smaller than RFP+ U87MG/GFP+ SR4987 xenografts as well (*p*<0.02, *p*<0.05, and *p*<0.001, respectively; Student's *t*-test). Fluorescence microscopy showed that, in the context of the tumor, the GFP+ SR4987PTX cells arranged themselves in strands and columns to form nets that entrapped the RFP+ U87MG cells (Fig. S7C). Conversely, the GFP+ SR4987 that had not been primed with PTX were intermingled with the U87MG cells without any propensity to organize into secondary structures (Fig. S7C). Histological examination revealed that the xenografts containing SR4987 cells, irrespective of their PTX priming, did not develop foci of necrosis, which are typically seen in the U87MG xenografts at the 4 and 6 week survival time (Fig. S7D). Both the size and histological appearance of U87MG and U87MG/SR4987 xenografts did not differ significantly from those xenografts generated by injection of the virus transduced RFP+ U87MG cells and RFP+ U87MG/GFP+ SR4987, respectively (data not shown).

## Discussion

The mouse stromal cell line SR4987 primed first *in vitro* with DXR and then co-cultured with HSCs results in a strong inhibition of CFU formations [14]. Noting this property, we wondered if hMSCs and SR4987 primed with an anti-cancer drug could acquire anti-tumor activity and consequently, be used for cancer therapy.

In order to validate this hypothesis, both mouse SR4987 and hMSCs were primed with PTX, because it demonstrated both strong anti-tumor [15], [25] and anti-angiogenic activities [16], [23]. Once released by primed cells, PTX could affect both tumor and ECs proliferation.

Initial experiments demonstrated that PTX was able to inhibit hMSCs and SR4987 proliferation but was not cytotoxic; up to 10,000 ng/ml did not affect cell viability. This PTX concentration was approximately 500 times higher than that necessary to produce an IC_50_ on MOLT-4, a human leukemia cell line very sensitive to PTX activity [20] that we used for monitoring the hMSCsPTX-CM and SR4987PTX-CM activity. Based on a previous study [14], we thus established a standard protocol to prime hMSCs and SR4987 with PTX, consisting of treating 3×10^5^ adherent cells with 2.000 ng/ml of PTX for 24 h in culture. In summary, we found that: a) hMSCs were able to rapidly incorporate PTX as confirmed by using PTX-F; b) hMSCsPTX can slowly release PTX, at least in part, in the culture medium in a time dependent manner, as confirmed by the HPLC analysis; c) formalin fixed hMSCs and were not effective to uptake PTX; d) both hMSCsPTX and SR4987PTX acquired a potent anti-tumor and anti-angiogenic activity *in vitro* that was dose dependent, and demonstrable by using their CM or by co-culture assay; e) hMSCsPTX co-injected in immunodeficient mice with human DU145 and SR4987PTX co-injected with U87MG or mouse B16 melanoma, significantly delayed tumor takes and reduced tumor growth. In the B16 melanoma model we observed that mouse stromal SR4987 cells are able “per se” to inhibit tumor cell proliferation at a level similar to SR4987PTX. This finding seems to be in agreement with the contradictory data reported from the literature that suggest both the capacity of mesenchymal cells and stromal cells to favour or to inhibit tumor progression [26]. In particular, the B16 tumor model represents a condition in which murine stromal cells anti-tumor activity has been demonstrated [27]. In this condition it may be that PTX loaded SR4987 cells increase their basal antitumor efficacy without a significant measurable effect.

Independently of this finding, we can conclude that our results, taken together, strongly support our initial hypothesis proposing even hMSCs as a carrier for chemotherapeutic drugs in human. In our opinion, our results disclose important new insights into functional properties of mammalian MSCs. From a toxicological view point, our results support the idea that mammals, at least within the BM stroma compartment, contain cells that may play a dual role in controlling drug toxicity. These cells may reduce or improve drug toxicity, depending on their capacity “to adsorb” and subsequently to release a drug even, as shown here for PTX, in its original form. This occurrence *in vivo* remains to be clarified. However, data from cancer patients receiving very high doses of chemotherapy seem to support this hypothesis [28]. Moreover, since MSCs and stromal cells have been isolated in many other mammalian tissues [29], it would be interesting to investigate whether MSCs derived from different tissues behave similarly to those in the BM. This may help to understand the causes that lead to organ specific toxicity by some chemotherapeutic agents in cancer patients [30].

It is also important to note that MSCs represent only a subpopulation of the more complex stromal cells population in a tissue [31]. Here, we have used hMSCs which express all the phenotypic and functional features that distinguish the MSCs phenotype from other stromal cells and a established mouse stromal cell line that express significant features of stemness [32]. We did not investigate whether other kinds of stromal cells primed with PTX behave like hMSCs and SR4987. Due to their sensitivity to PTX, ECs, as shown here and by other authors [16], can not be used for PTX loading. Other reports show that PTX is a strong inhibitor of fibroblasts and smooth muscle cell proliferation *in vitro* but do not investigate its toxicity [33], [34].

The mechanism of PTX uptake by cells and its binding to microtubules has been extensively studied [21], [35]. In this study we observed that PTX, upon entering the cells, locates into vesicles derived from Golgi apparatus. However, very little is known about the cells capacity to release the PTX or about the chemotherapy resistance of MSCs [18], [36]. A report from Chaudhary et al, demonstrated that multidrug efflux pump in BM derived HSCs involved P-gp, a mechanism claimed for multidrug resistance (MDR) in cancer cells [37], [38]. P-glycoprotein (P-gp) is a 170 Kd membrane molecule that has been described in tumor cells, where it contributes to the multidrug resistance (MDR) phenomenon by promoting efflux of multiple, structurally unrelated anticancer drugs. P-gp is very sensitive to Verapamil which is an important specific first-generation efflux pump inhibitor [39]. In our investigations, we observed that both hMSCs and SR4987 express very high levels of P-gp and that treatment with PTX and VP down regulates P-gp expression (Fig. S3). The modulation of PTX sensitivity significantly exerted by VP on SR4987 may suggest that PTX, as well other lypophilic cytotoxic drugs which bind tubulin, such as colchicine, colcemid, vinblastine and vincristine, may be secreted through the P-gp system [40]–[43]. Preliminary experiments performed with SR4987 on uptake/release of PTX in the presence of VP confirm this mechanism. However, because PTX sensitivity of hMSCs was not very much affected by VP treatment, we can not exclude that, besides P-gp in hMSCs, other mechanisms of MDR may be involved.

Despite the mechanism in which hMSCs release PTX, our study demonstrates for the first time that, through a simple *in vitro* process of priming, hMSCs incorporate a sufficient amount of PTX. Despite a loss of cells due to apoptosis, the majority of them (80%) remain alive, are unable to proliferate and, once located in the vicinity of both cancer cells or ECs, can release the drug in a time dependent manner and in a quantity sufficient enough to inhibit proliferation *in vitro* and to reduce dramatically both human and mouse tumor growth *in vivo*. Because the administration of free PTX to cancer cells did not affect their growth, it suggests that hMSCsPTX *in vivo* may slowly release the drug as they do *in vitro*, thus determining a PTX concentration sufficient to affect cancer cell proliferation.

In summary, to our knowledge this is the first demonstration that hMSCs can be loaded *in vitro* with a chemotherapeutic drug and used for cancer treatment *in vivo*. Since priming hMSCs with PTX is a simple procedure that neither requires any genetic manipulation of cells nor much time, we are confident that, once the effectiveness of hMSCsPTX on established cancers in mice is proven, our procedure could be applied in cancer patients, perhaps in combination with other methods, which use, for example, nanoparticles or liposome for drug transport.

## Materials and Methods

### Ethics Statement

All animal experiments were performed according to international law and policies (EEC C.D.86/609,OJL358,1987; Guide for the Care and Use of Laboratory Animals, U.S. National Research Council, 1996). Experiments and care/welfare were performed in an animal facility with protocols authorized by Ministero della Salute (D.I.116/1992,Circ.8/1994, D.M. 60/2003-A, P.C.ID:1/2009).

### Tumor cells

For the *in vitro* and *in vivo* studies three human tumor cell (TC) lines were used: MOLT-4 (acute lymphoblastic leukemia) [44]; T98G (glioblastoma) [45]; DU-145 (prostate carcinoma) [46] and B16 mouse melanoma [47]. *In vivo* studies have also been performed by using RFP+U87MG glioblastoma cell. (For details see supporting information text S1).

### MSCs expansion and characterization

HMSCs were prepared as previous described [48] from the mononuclear cell fraction of human bone marrow purchased from Lonza (USA) who manage human tissue in accordance with current US regulations governing tissue banking in the Code of Federal Regulations (CFR), 21 CFR Part 1271. After expansion, cells were characterized for CD markers expression, differentiating capacity (osteo-condro-adipocytes) [49] (See supporting information Text S1).

### Murine stromal cells

For murine stromal cells, a cell line established from bone marrow (SR4987) having a high degree of stemness was used. It is positive for some stem cells markers as vimentin, CD44+, CD73+,CD105+, CD106+, Sca-1+, CD34+, contain 50% CD45+ cells and is capable of differentiating into osteocyte and chondrocytes [17], [50]. For some specific *in vivo* studies, transfected cells (GFP+SR4987) have been prepared (see Suppoprting Information Text S1). P-glycoprotein (P-gp) expression was evaluated by using a mouse monoclonal antibodies anti-human P-gp (clone C1, Ylem, Italy). Cell were then analyzed by FACS.

### Procedure for drug uptake and release by MSCs

SR4987 and hMSCs (3×10^5^) were treated 24 hours with PTX (2.000 ng/ml) (Serva, Germany). At the end of the incubation, the cells were washed twice with PBS, then trypsinized, washed twice in HBSS and seeded in a new flask. After 24 h of culture, the cell conditioned medium (CM) was collected and replaced by repeating this procedure at 48, 72, 96 and 144 hours. The CM were tested for their anti-tumor proliferation activity *in vitro* by using CM from untreated MSCs as negative controls. The passive membrane drug adsorption and release by fixed cells were also verified. The internalisation of PTX into MSCs was checked by Fluorescent PTX (Oregon Green 488 Taxol, Invitrogen, UK). The effect of 24 h drug treatment on the cell cycle of MSCs was evaluated by FACS. The presence of PTX in the CM of primed MSCs was confirmed by HPLC [51] (see supporting information Text S1).

### *In vitro* anti proliferative assay on tumor cell lines

The effect of both PTX and CM from PTX-treated MSCs (MSCsPTX-CM) on TC proliferation was studied in 96 multiwell plates (Sarstedt, Germany) on MOLT-4, T98G and DU145 cells according to a MTT (3-(4,5-dimethyl-2-thiazolyl)-2,5-diphenyl-2-H-tetrazoliumbromide) assay [52]. The inhibitory concentrations (IC_50_ and IC_90_) were determined according to the Reed and Muench formula [53]. The antitumoral activity of MSCsPTX-CM was compared to that of pure PTX and expressed as PTX equivalent concentration (PEC) according to the following algorithm: PEC (ng/ml) = DF_50_CM×IC_50_PTX (DF_50_CM is the dilution factors (DF) at which the 50% of inhibition was observed with MSCsPTX-CM; IC_50_PTX is the concentration (ng/ml) of pure PTX producing 50% of inhibition). The PTX release (PR) by a single primed MSC was calculated as ratio between the PEC and the number of cells seeded: PR (pg/cell) = PEC (ng/ml)×CM volume (ml)/number of cell seeded (see supporting information Text S1).

### *In vitro* inhibition of tumor growth by MSCsPTX

To verify the ability of MSCsPTX to inhibit *in vitro* TC proliferation, a co-culture system was applied by mixing TCs with different amounts of MSCs to final ratios of MSCs/TCs of 1-10-100. Arbitrary values of IC_50_ and IC_90_ were calculated as ratio MSCs/TCs able to inhibit respectively 50% and 90% of TC proliferation (see supporting information Text S1).

### Evaluation of anti-angiogenic properties of SR4987 and hMSCs primed with PTX *in vitro*

The anti-angiogenic potential of the CM, from MSCs primed or not with PTX, was tested on the proliferation of Human Umbilical vein ECs (HUVEC) (purchased from Lonza) and on human derma microvascular ECs (HMECs) isolated and maintained as previously described [54]. Both ECs phenotypes were routinely maintained in EGM bullet kit plus 10% FCS (Lonza). The rat aorta ring assay was also performed to investigate the anti-angiogenic potential of control and MSCsPTX-CM according to the procedure previously described [24], [55], [56]. Quantification of angiogenesis was obtained by taking photographs every three days and by counting the number of microvessels arising from aorta rings [24] (see supporting information Text S1).

### Evaluation of anti-tumor activity of hMSCsPTX and mouse SR4987PTX *in vivo*

The capacity of control hMSCs and hMSCsPTX to affect DU145 and B16 melanoma growth were performed on eight-week-old male NOD/SCID mice (JAX Mice: NOD.CB17-Prkdc scid/j) and Nude mice (4-week-old male athymic nude-Foxn1 nu/nu from Harlan, Italy) respectively. The subcutaneous grafting of RFP+ U87MG glioblastoma cells mixed with GFP+ SR4987 cells primed or not with PTX was also performed in nude athymic mice. Eight- week-old female mice C57Bl6 (Charles River, Italy) were used to evaluate the effect of mouse control SR4987 and SR4987-PTX on B16 melanoma (see supporting information Text S1).

## Supporting Information

Figure S1 **Characterization of Mesenchymal Stem Cells expanded from human bone marrow.** The MSC feature of the expanded cell population has been confirmed by their capacity to differentiate into adipocyte (A), osteoblasts (B) and condhroblasts (C) under specific stimulation. Figure 1D shows unstimulated MSCs (Negative Control). The box on the right shows the pattern of CD expression that is confirmed to be typical of MSCs.
(TIF)

Figure S2 **Cell cycle analysis by FACS.** The histograms show the effects of PTX treatment on hMSCs and SR4987 cell cycle after 24 hours of treatment. The percentages of cells counted in each different cell phase (G_0_/G_1_, S and G_2_/M ) are reported and compared to these found in untreated cells (CTRL). PTX 24 h = cells after 24 h of PTX treatment; PTX 24+24 h = PTX treated cells, then subcultured for 24 h without PTX ; PTX 24+72 h = PTX treated cells, then subcultured for 72 h without PTX. Under the abscissa are reported the percentages of cell viability evaluated by Trypan Blue.
(TIF)

Figure S3 **Modulation of P-gp expression and PTX sensitivity.** (A) The histogram shows the basal expression of P-gp by hMSCs and SR4987 (CTRL), its modulation after 24 hours of treatment with 2 µg/ml paclitaxel (PTX+) or 20 uM Verapamil (VP+). P-gp expression was evaluated by FACS and reported as ratio between fluorescence intensity measured on cells treated with specific labelled antibody and that of cells treated with isotype control antibody. (B) The histogram shows the IC_50_ values (ng/ml) for PTX determined in a antiproliferation MTT assay in the absence and in the presence of 20 µM VP. Each point reports the mean value of three independent experiments.
(TIF)

Figure S4 **Internalization and kinetics of Fluorescent PTX (PTX-F) decrease in hMSCs.** The histogram demonstrates that PTX-F was internalized by hMSCs. The study was conducted by treating hMSCs with PTX-F for 24 hrs. Thereafter, the cells were harvested by trypsin and washed, and then analysed by FACS immediately (time 0) at 8 and 24 hours while maintained resuspended in PBS. Violet histogram = cells treated with unlabelled Taxol; Green histogram = cells treated with FITC-conjugated Taxol; X axis = green fluorescence intensity; Y axis = number of cells.
(TIF)

Figure S5 **Mouse SR4987PTX-CM inhibit HUVECs and HMECs proliferation.** HUVECs (A) and (C) and HMECs (B) were cultured for 72 hrs in the presence or in the absence of control SR4987-CM and SR4987PTX-CM at different dilutions. SR4987PTX-CM at 1∶2 and 1∶4 dilutions were cytotoxic for HUVECs, while they induced a significant growth inhibition on HMECs. In C photographs (20× magnifications) show the cytotoxic effect of SR4987PTX-CM on HUVECs at 1∶2 and 1∶4 dilutions. Control SR4987-CM do not inhibit, but even seem to improve proliferation of both HUVECs and HMECs. The values in A and B are the means ± SD of two different experiments *p<0.05, *p<0.01 vs* untreated MMSCs.
(TIF)

Figure S6 **Mouse SR4987PTX inhibit B16 melanoma growth in syngeneic C57Bl6 mice.** The Figure shows the effect of control SR4987 and primed SR4987PTX (0.4×10^5^) mixed at 1∶5 ratio with B16 melanoma cells (2×10^5^) and injected s.c into syngeneic C57Bl6 mice (see also Table 1). In (A) SR4987PTX induced a significant reduction of B16 tumor volume calculated by measuring tumor diameters with a calibre. Interesting, even the co-injection of control SR4987 with B16 melanoma cells reduced the tumor volumes. In (B) the effect of SR4987PTX on B16 tumor appearance showing a significative delay in tumor takes. In (C) the photo of B16 tumors removed from the s.c of control, SR4987PTX and SR4987 treated mice at the time of mice sacrifice. * p<0.05 and ** p<0.01 vs control B16 volume.
(TIF)

Figure S7 **Effects of GFP+ SR4987-PTX on the growth and histology of subcutaneous RFP+U87MG xenografts.** At the 2, 4, and 8 week survival time, the tumor xenografts generated by co-injection of RFP+U87MG glioblastoma cells and GFP+SR4987-PTX cells showed significantly smaller diameter compared with tumor generated by injection of glioblastoma cells or by co-injection of RFP+U87MG and GFP+SR4987 cells (* *p*<0.05; ** *p*<0.02; *** *p*<0.001) (A). At two weeks after grafting: the histology of RFP+U87MG/GFP+SR4987-PTX xenografts shows reduced cell density with regions of Matrigel that are not colonized by the tumor cells and vimentin expressing cells arranged in columns (B). At 4 weeks after grafting : RFP+ U87MG xenograft and RFP+ U87MG/GFP+ SR4987 xenograft show green MSCs intermingled with the red glioblastoma cells. RFP+ U87MG/GFP+ SR4987-PTX xenograft shows that the green MSCs arrange themselves to form septi encircling the red glioblastoma cells (C). At 6 weeks after injection, tumor xenografts containing GFP+SR4987 do not develop areas of necrosis that are a typical feature of the RFP+U87MG xenografts at this time point (D).
(TIF)

Table S1 **Evaluation of apoptosis in hMSCs and SR-4987 after PTX treatment.** The evaluation of apoptosis was performed by flow cytometry by the Annexin-V binding assay and confirmed by the quantification of the sub-G1 cell population. Data report the percentage of apoptotic/necrotic cells (mean ± s.d. of three assays). The assays were performed with different passages of SR4987 and different bone marrow donors). CTRL = Untreated cells; PTX 24 h = cells after 24 h of PTX treatment; PTX 24+24 h = treated cells, then subcultured for 24 h without PTX.
(DOC)

Text S1

## References

[1] Weldon JE, Xiang L, Chertov O, Margulies I, Kreitman RJ (2009). A protease-resistant immunotoxin against CD22 with greatly increased activity against CLL and diminished animal toxicity.. Blood.

[2] Dhar S, Gu FX, Langer R, Farokhzad OC, Lippard SJ (2008). Targeted delivery of cisplatin to prostate cancer cells by aptamer functionalized Pt (IV) prodrug-PLGA-PEG nanoparticles.. Proc Natl Acad Sci USA.

[3] Loebinger MR, Eddaoudi A, Davies D, Janes SM (2009). Mesenchymal stem cell delivery of TRAIL can eliminate metastatic cancer.. Cancer Res.

[4] Nakamura K, Ito Y, Kawano Y, Kurozumi K, Kobune M (2004). Antitumor effect of genetically engineered mesenchymal stem cells in a rat glioma model.. Gene Ther.

[5] Menon LG, Shi VJ, Carroll RS (2009). Mesenchymal stromal cells as a drug delivery system, StemBook, ed.. The Stem Cell Research Community.

[6] Nakamizo A, Marini F, Amano T, Khan A, Studeny M (2005). Human bone marrow-derived mesenchymal stem cells in the treatment of gliomas.. Cancer Res.

[7] Stagg J, Lejeune L, Paquin A, Galipeau J (2004). Marrow stromal cells for interleukin-2 delivery in cancer immunotherapy.. Hum Gene Ther.

[8] Kucerova L, Altanerova V, Matuskova M, Tyciakova S, Altaner C (2007). Adipose tissue-derived human mesenchymal stem cells mediated prodrug cancer gene therapy.. Cancer Res.

[9] Studeny M, Marini FC, Champlin RE, Zompetta C, Fidler IJ (2002). Bone marrow-derived mesenchymal stem cells as vehicles for interferon-ß delivery into tumors.. Cancer Res.

[10] Izadpanah R, Trigg C, Patel B, Kriedt C, Dufour J (2006). Biologic properties of mesenchymal stem cells derived from bone marrow and adipose tissue.. J Cell Biochem.

[11] Yong RL, Shinojima N, Fuevo J, Gumin J, Vecil GG (2009). Human bone marrow-derived mesenchymal stem cells for intravascular delivery of oncolytic adenovirus Delta24-RGD to human gliomas.. Cancer Res.

[12] Elzaouk L, Moelling K, Pavlovic J (2006). Anti-tumor activity of mesenchymal stem cells producing IL-12 in a mouse melanoma model.. Exp Dermatol.

[13] Zhang XB, Beard BC, Trobridge GD, Wood BL, Sale GE (2008). High incidence of leukemia in large animals after stem cell gene therapy with a HOXB4-expressing retroviral vector.. J Clin Invest.

[14] Pessina A, Piccirillo M, Mineo E, Catalani P, Gribaldo L (1999). Role of SR4987 stromal cells in the modulation of doxorubicin toxicity to in vitro granulocyte-macrophage progenitors (CFU-GM).. Life Sci.

[15] Schiff PB, Fant J, Horwitz SB (1979). Promotion of microtubule assembly in vitro by taxol.. Nature.

[16] Belotti D, Vergani V, Drudis T, Borsotti P, Pitelli MR (1996). The microtubule-affecting drug paclitaxel has antiangiogenic activity.. Clin Cancer Res.

[17] Pessina A, Mineo E, Neri MG, Gribaldo L, Colombi R (1992). Establishment and characterization of a new murine cell line (SR4987) derived from marrow stromal cells.. Cytotechnology.

[18] Li J, Law HKW, Lau YL, Chan GCF (2004). Differential damage and recovery of human mesenchymal stem cells after exposure to chemotherapeutic agents.. British J Haematol.

[19] Polioudaki H, Kastrinaki MC, Papadaki HA, Theodoropoulos PA (2009). Microtubule-interacting drugs induce moderate and reversible damage to human bone marrow mesenchymal stem cells.. Cell Prolif.

[20] Zhang P, Tao DD, Feng YD, Xie DX, Zhou JF (2006). Paclitaxel induces apoptosis of acute leukaemia cells in S phase.. Ai Zheng.

[21] Molinari A, Calcabrini A, Meschini S, Stringaro A, Del Bufalo D (1998). Detection of P-glycoprotein in the Golgi apparatus of drug-untreated human melanoma cells.. Int J Cancer.

[22] Kumar G, Ray S, Walle T, Huang Y, Willigham M (1995). Comparative in vitro cytotoxic effects of taxol and its major human metabolite 6 alpha-hydroxytaxol.. Cancer Chemother Pharmacol.

[23] Kunstfeld R, Wickenhauser G, Michaelis U, Teifel M, Umek W (2003). Paclitaxel encapsulated in cationic liposomes diminishes tumor angiogenesis and melanoma growth in a “humanized” SCID mouse model.. J Invest Dermatol.

[24] Nicosia RF, Ottinetti A (1990). Growth of microvessel in serum-free matrix culture of rat aorta: a quantitative assay of angiogenesis in vitro.. Lab Invest.

[25] Rennison ME, Handel SE, Wilde CJ, Burgoyne RD (1992). Investigation of the role of microtubules in protein secretion from lactating mouse mammary epithelial cells.. J Cell Sci.

[26] Ciavarella S, Dominici M, Dammacco F, Silvestris F (2011). Mesenchymal Stem Cells: A New Promise in Anticancer Therapy.. Stem Cells Develop.

[27] Maestroni GJM, Hertens E, Galli P (1999). Factor(s) from non-macrophage bone marrow stromal cells inhibit Lewis lung carcinoma and B16 melanoma growth in mice.. Cell Mol Life Sci.

[28] Breeden JH, Vollmer JT, Twomey PL (1982). Toxicity of very high dose nitrosourea administration.. Cancer.

[29] Kern S, Eichler H, Stoeve J, Klüter H, Bieback K (2006). Comparative analysis of mesenchymal stem cells from bone marrow, umbilical cord blood, or adipose tissue.. Stem Cells.

[30] Gille L, Nohl H (1997). Analyses of the molecular mechanism of adriamycin-induced cardiotoxicity.. Free Radic Biol Med.

[31] Tormin A, Brune JC, Olsson E, Valcich J, Neuman U (2009). Characterization of bone marrow-derived mesenchymal stromal cells (MSC) based on gene expression profiling of functionally defined MSC subsets.. Cytotherapy.

[32] Comite P, Cobianchi L, Avanzini MA, Zonta S, Mantelli M (2010). Isolation and ex vivo expansion of bone marrow-derived porcine mesenchymal stromal cells: potential for application in an experimental model of solid organ transplantation in large animals.. Transplant Proc.

[33] Zhang J, Melhem M, Kassing W, Kelly B, Wang Y (2007). In vitro paclitaxel and radiation effects on the cell types responsible for vascular stenosis: a preliminary analysis.. Blood Purif.

[34] Axel DI, Kunert W, Göggelmann C, Oberhoff M, Herdeg C (1997). Paclitaxel inhibits arterial smooth muscle cell proliferation and migration in vitro and in vivo using local drug delivery.. Circulation.

[35] Singla AK, Garg A, Aggarwal D (2002). Paclitaxel and its formulations.. Int J Pharm.

[36] Mueller LP, Luetzkendorf J, Mueller T, Reichelt K, Simon H (2006). Presence of mesenchymal stem cells in human bone marrow after exposure to chemotherapy: evidence of resistance to apoptosis induction.. Stem Cells.

[37] Kane SE, Pastan I, Gottesman MM (1990). Genetic basis of multidrug resistance of tumor cells.. J Bioenerg Biomembr.

[38] Chaudhary PM, Roninson IB (1993). Induction of multidrug resistance in human cells by transient exposure to different chemotherapeutic drugs.. J Natl Cancer Inst.

[39] Nobili S, Landini I, Giglioni B, Mini E (2006). Pharmacological strategies for overcoming multidrug resistance.. Curr Drug Targets.

[40] Lalande ME, Ling V, Miller RG (1981). Hoechst 33342 dye uptake as a probe of membrane permeability changes in mammalian cells.. Proc Natl Acad Sci USA.

[41] Lampidis TJ, Munck JN, Krishan A, Tapiero H (1985). Reversal of resistance to rhodamine 123 in adriamycin-resistant Friend leukemia cells.. Cancer Res.

[42] Neyfakh AA (1988). Use of fluorescent dyes as molecular probes for the study of multidrug resistance.. Exp Cell Res.

[43] Neyfakh AA, Dmitrevskaya TV, Serpinskaya AS (1988). The membrane transport system responsible for multidrug resistance is operating in nonresistant cells.. Exp Cell Res.

[44] Minowada J, Onuma T, Moore GE (1972). Rosette-forming human lymphoid cell lines. I. Establishment and evidence for origin of thymus-derived lymphocytes.. J Natl Cancer Inst.

[45] Stein G (1979). T98G: an anchorage-independent human tumor cell line that exhibits stationary phase G1 arrest in vitro.. J Cell Physiol.

[46] Mickey DD, Stone KR, Wunderli H, Mickey GH, Vollmer RT (1977). Heterotransplantation of a human prostatic adenocarcinoma cell line in nude mice.. Cancer Res.

[47] Riley V (1963). Enzymatic determination of transmissible replicating factors associated with mouse tumors.. Ann N Y Acad Sci.

[48] Pessina A, Eletti B, Croera C, Savalli N, Diodovich C (2004). Pancreas developing markers expressed on human mononucleated umbilical cord blood cells.. Biochem Biophys Res Commun.

[49] Pittenger MF, Mackay AM, Beck SC, Jaiswal RK, Douglas R (1999). Multilineage potential of adult human mesenchymal stem cells.. Science.

[50] Pessina A, Sisto F, Coccè V, Cavicchini E, Ciusani E (2011). A mesenchymal stromal cell line resistant to paclitaxel that spontaneously differentiates into osteoblast-like cells.. Cell Biol Toxicol.

[51] Gianni L, Kearns CM, Giani A, Capri G, Vigano L (1995). Nonlinear pharmacokinetic of paclitaxel and its pharmacokinetic/pharmacodynamic relationships in humans.. J Clin Oncol.

[52] Mossman T (1983). Rapid colorimetric assay for cellular growth and survival: application to proliferation and cytotoxicity assays.. J Immunol Methods.

[53] Reed LJ, Muench H (1938). A simple method of estimating fifty percent endpoints.. The American Journal of Hygiene.

[54] Caruso A, Rotola A, Comar M, Favilli F, Galvan M (2002). HHV-6 infects human aortic and heart microvascular endothelial cells increasing their ability to secrete proinflammatory chemokines.. J Med Virol.

[55] Elsdale T, Bard J (1992). Collagen substrata for study of cell behavior.. J Cell Bio.

[56] Invernici G, Emanueli C, Madeddu P, Cristini S, Gadau S (2007). Human fetal aorta contains vascular progenitor cells capable of inducing vasculogenesis, angiogenesis, and myogenesis in vitro and in a murine model of peripheral ischemia.. Am J Pathol.

