# Supplementary figures and images for: Mesenchymal Stromal Cells Primed with Paclitaxel Provide a New Approach for Cancer Therapy
Source: PLoS One. 2011 Dec 20;6(12):e28321. doi: 10.1371/journal.pone.0028321 (PMC3243689; doi:10.1371/journal.pone.0028321)

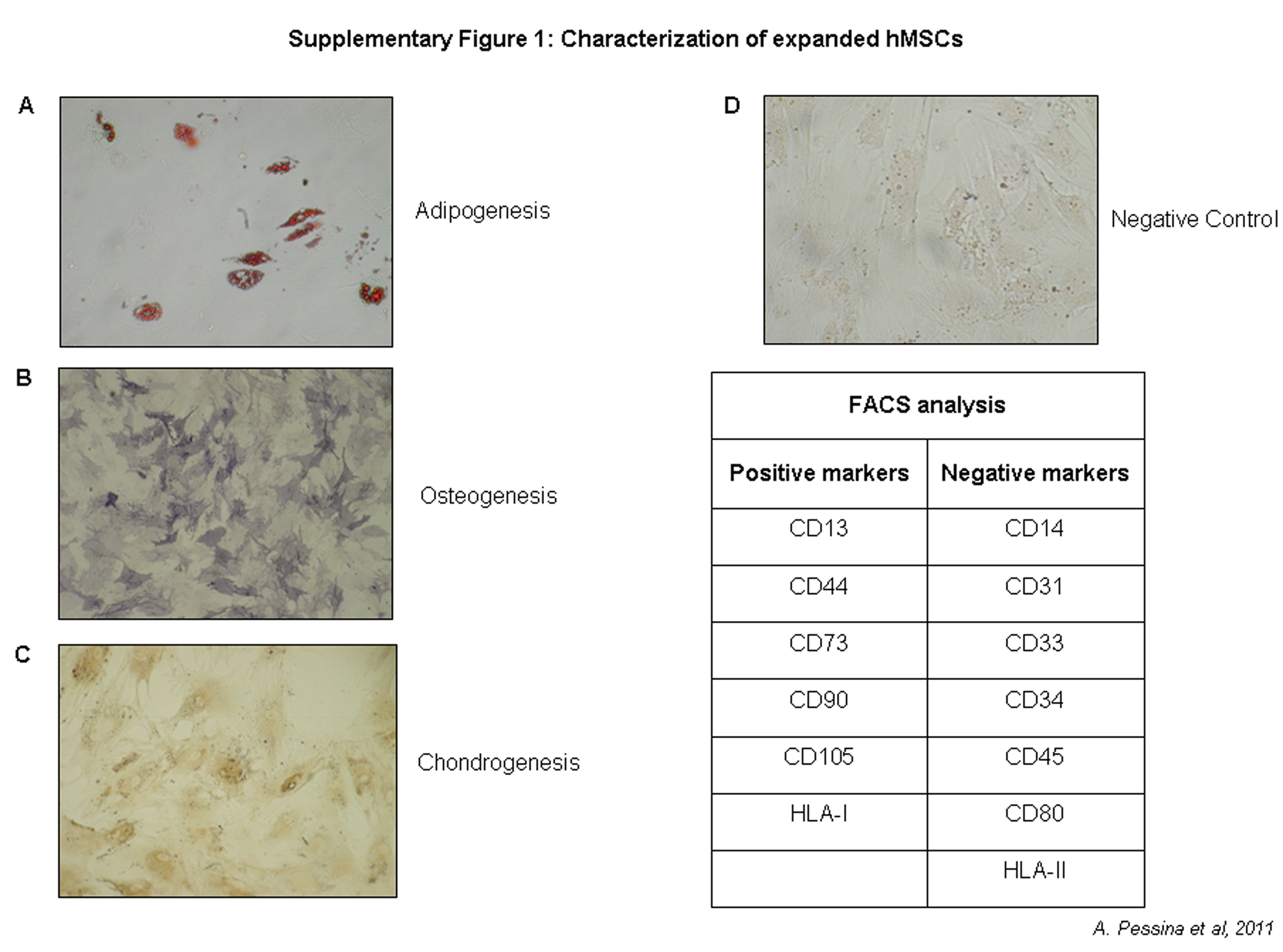

Supplement: Figure S1 — Characterization of Mesenchymal Stem Cells expanded from human bone marrow. The MSC feature of the expanded cell population has been confirmed by their capacity to differentiate into adipocyte (A), osteoblasts (B) and condhroblasts (C) under specific stimulation. Figure 1D shows unstimulated MSCs (Negative Control). The box on the right shows the pattern of CD expression that is confirmed to be typical of MSCs. (TIF) [file pone.0028321.s001.tif]

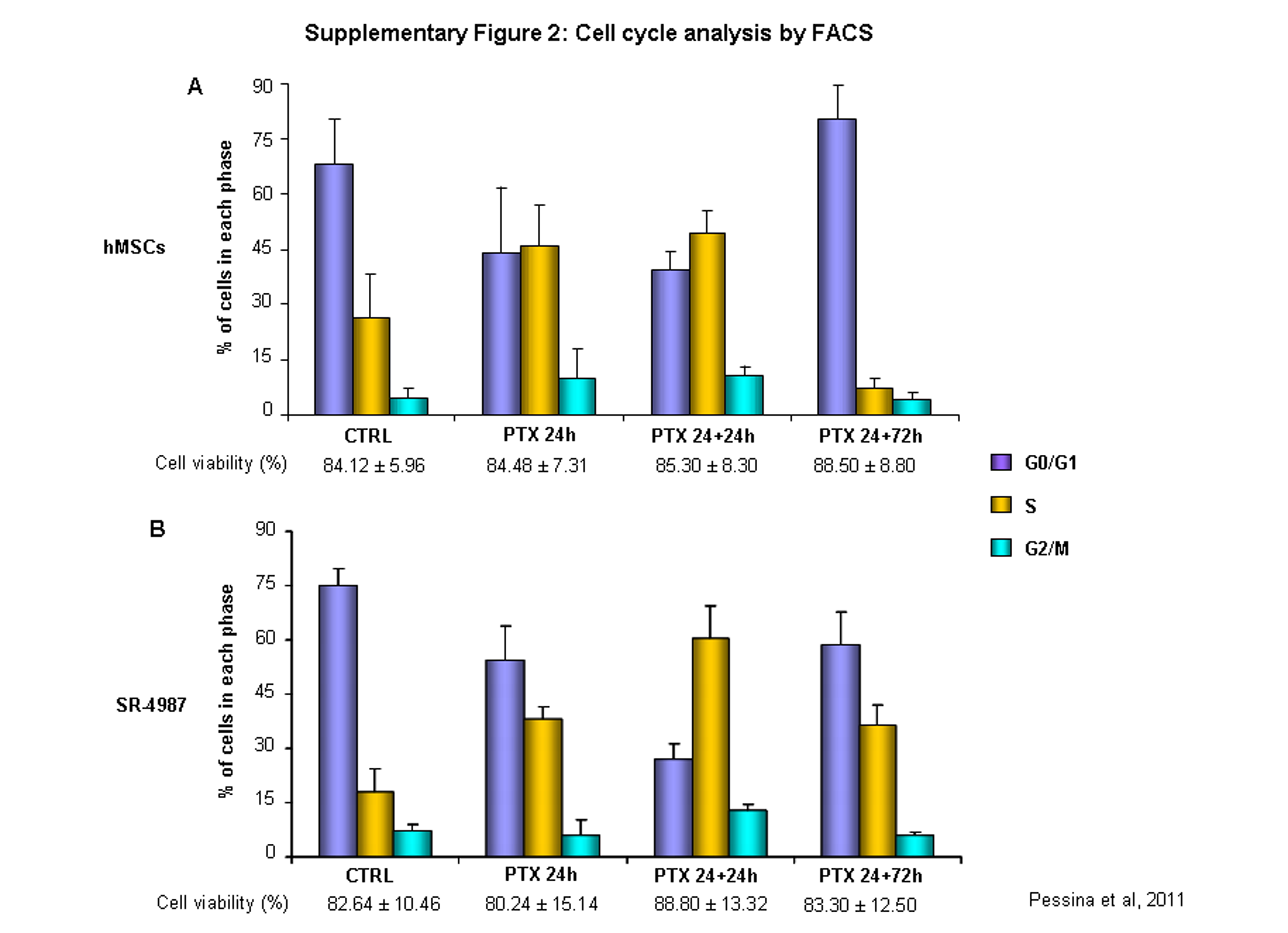

Supplement: Figure S2 — Cell cycle analysis by FACS. The histograms show the effects of PTX treatment on hMSCs and SR4987 cell cycle after 24 hours of treatment. The percentages of cells counted in each different cell phase (G0/G1, S and G2/M ) are reported and compared to these found in untreated cells (CTRL). PTX 24 h = cells after 24 h of PTX treatment; PTX 24+24 h = PTX treated cells, then subcultured for 24 h without PTX ; PTX 24+72 h = PTX treated cells, then subcultured for 72 h without PTX. Under the abscissa are reported the percentages of cell viability evaluated by Trypan Blue. (TIF) [file pone.0028321.s002.tif]

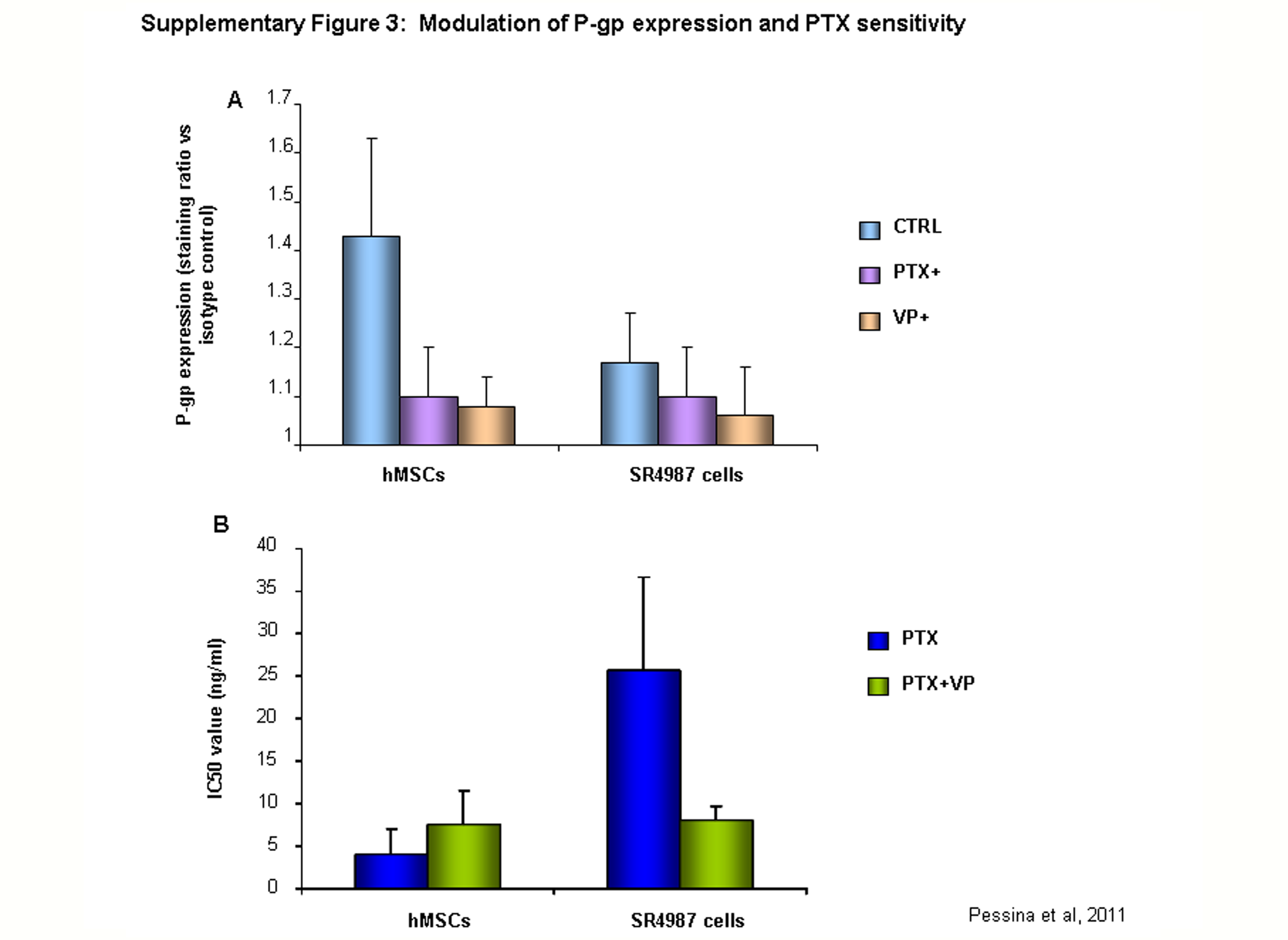

Supplement: Figure S3 — Modulation of P-gp expression and PTX sensitivity. (A) The histogram shows the basal expression of P-gp by hMSCs and SR4987 (CTRL), its modulation after 24 hours of treatment with 2 µg/ml paclitaxel (PTX+) or 20 uM Verapamil (VP+). P-gp expression was evaluated by FACS and reported as ratio between fluorescence intensity measured on cells treated with specific labelled antibody and that of cells treated with isotype control antibody. (B) The histogram shows the IC50 values (ng/ml) for PTX determined in a antiproliferation MTT assay in the absence and in the presence of 20 µM VP. Each point reports the mean value of three independent experiments. (TIF) [file pone.0028321.s003.tif]

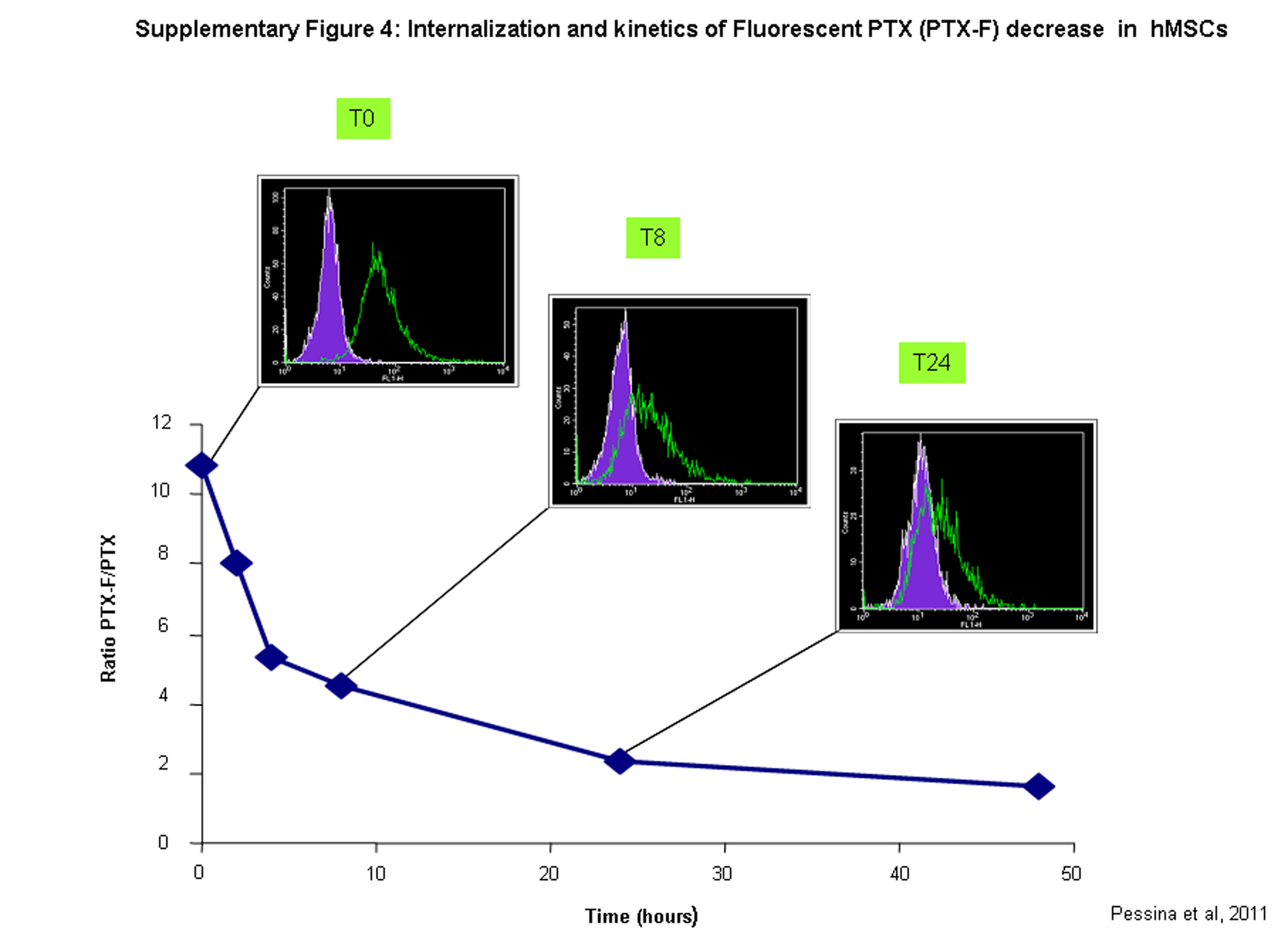

Supplement: Figure S4 — Internalization and kinetics of Fluorescent PTX (PTX-F) decrease in hMSCs. The histogram demonstrates that PTX-F was internalized by hMSCs. The study was conducted by treating hMSCs with PTX-F for 24 hrs. Thereafter, the cells were harvested by trypsin and washed, and then analysed by FACS immediately (time 0) at 8 and 24 hours while maintained resuspended in PBS. Violet histogram = cells treated with unlabelled Taxol; Green histogram = cells treated with FITC-conjugated Taxol; X axis = green fluorescence intensity; Y axis = number of cells. (TIF) [file pone.0028321.s004.tif]

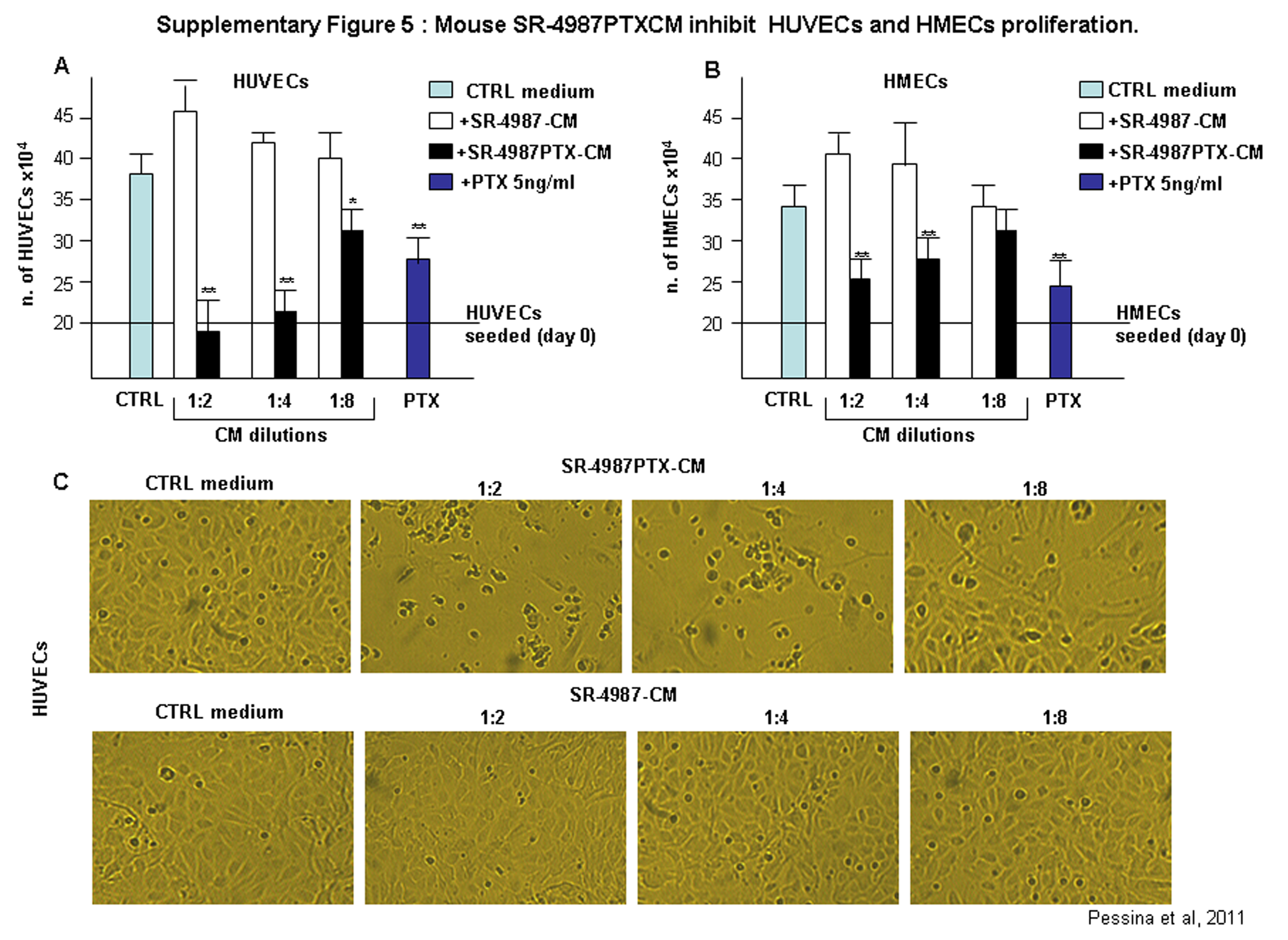

Supplement: Figure S5 — Mouse SR4987PTX-CM inhibit HUVECs and HMECs proliferation. HUVECs (A) and (C) and HMECs (B) were cultured for 72 hrs in the presence or in the absence of control SR4987-CM and SR4987PTX-CM at different dilutions. SR4987PTX-CM at 1∶2 and 1∶4 dilutions were cytotoxic for HUVECs, while they induced a significant growth inhibition on HMECs. In C photographs (20× magnifications) show the cytotoxic effect of SR4987PTX-CM on HUVECs at 1∶2 and 1∶4 dilutions. Control SR4987-CM do not inhibit, but even seem to improve proliferation of both HUVECs and HMECs. The values in A and B are the means ± SD of two different experiments *p<0.05, p<0.01 vs untreated MMSCs. (TIF) [file pone.0028321.s005.tif]

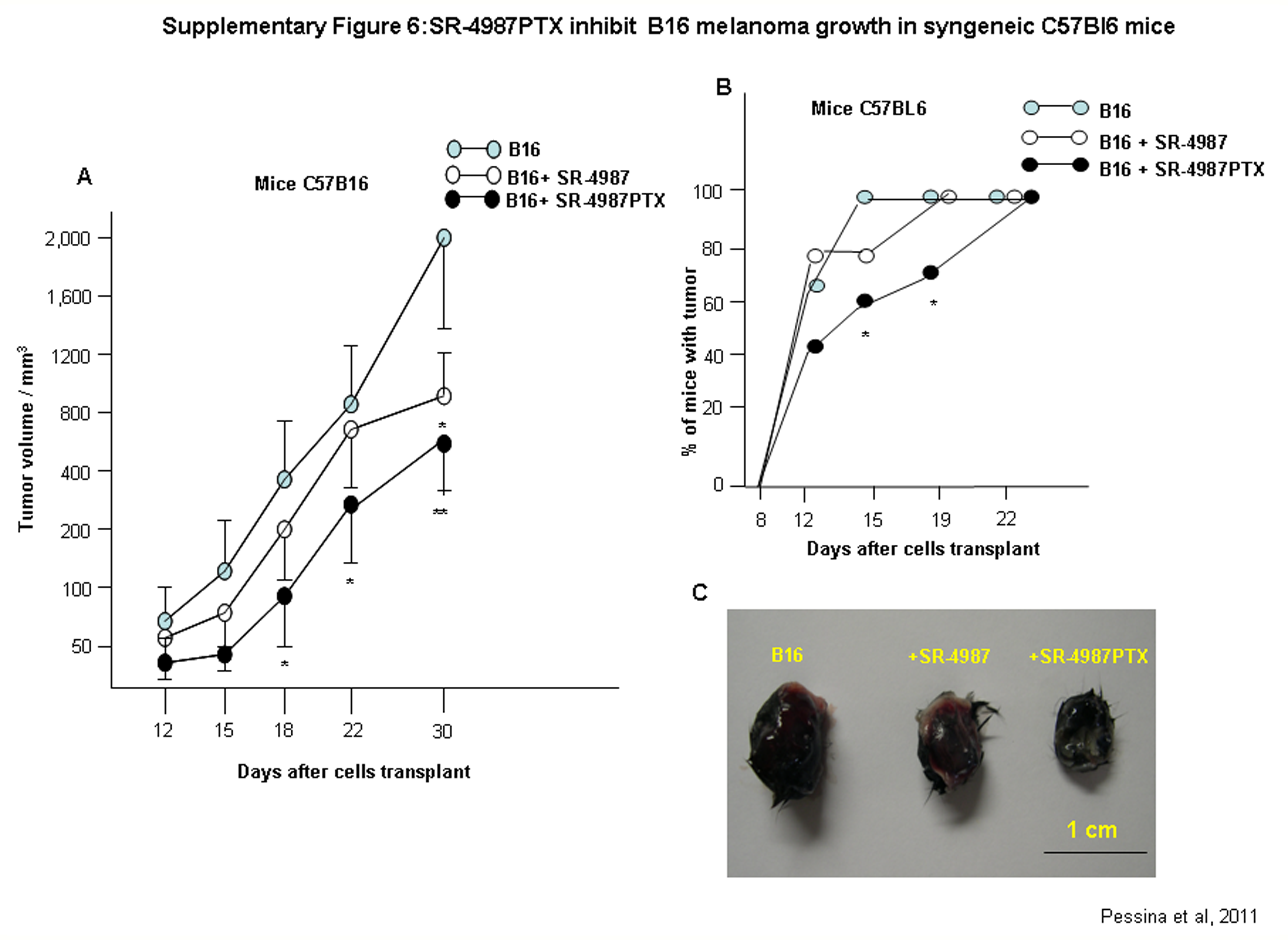

Supplement: Figure S6 — Mouse SR4987PTX inhibit B16 melanoma growth in syngeneic C57Bl6 mice. The Figure shows the effect of control SR4987 and primed SR4987PTX (0.4×10^5^) mixed at 1∶5 ratio with B16 melanoma cells (2×10^5^) and injected s.c into syngeneic C57Bl6 mice (see also Table 1). In (A) SR4987PTX induced a significant reduction of B16 tumor volume calculated by measuring tumor diameters with a calibre. Interesting, even the co-injection of control SR4987 with B16 melanoma cells reduced the tumor volumes. In (B) the effect of SR4987PTX on B16 tumor appearance showing a significative delay in tumor takes. In (C) the photo of B16 tumors removed from the s.c of control, SR4987PTX and SR4987 treated mice at the time of mice sacrifice. * p<0.05 and ** p<0.01 vs control B16 volume. (TIF) [file pone.0028321.s006.tif]

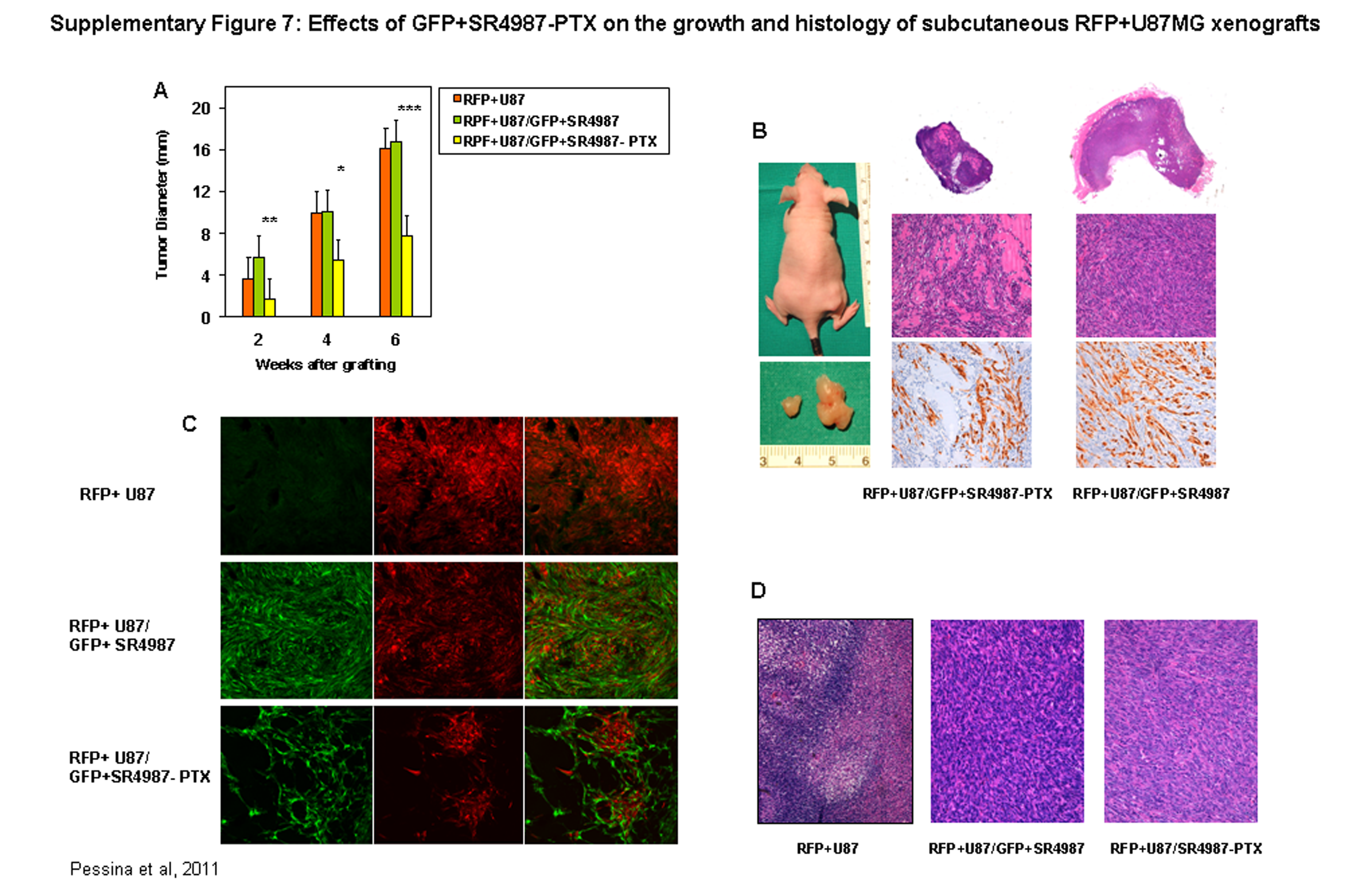

Supplement: Figure S7 — Effects of GFP+ SR4987-PTX on the growth and histology of subcutaneous RFP+U87MG xenografts. At the 2, 4, and 8 week survival time, the tumor xenografts generated by co-injection of RFP+U87MG glioblastoma cells and GFP+SR4987-PTX cells showed significantly smaller diameter compared with tumor generated by injection of glioblastoma cells or by co-injection of RFP+U87MG and GFP+SR4987 cells (* p<0.05; ** p<0.02; *** p<0.001) (A). At two weeks after grafting: the histology of RFP+U87MG/GFP+SR4987-PTX xenografts shows reduced cell density with regions of Matrigel that are not colonized by the tumor cells and vimentin expressing cells arranged in columns (B). At 4 weeks after grafting : RFP+ U87MG xenograft and RFP+ U87MG/GFP+ SR4987 xenograft show green MSCs intermingled with the red glioblastoma cells. RFP+ U87MG/GFP+ SR4987-PTX xenograft shows that the green MSCs arrange themselves to form septi encircling the red glioblastoma cells (C). At 6 weeks after injection, tumor xenografts containing GFP+SR4987 do not develop areas of necrosis that are a typical feature of the RFP+U87MG xenografts at this time point (D). (TIF) [file pone.0028321.s007.tif]
