# Supplementary material for: Mesenchymal Stromal Cells Primed with Paclitaxel Provide a New Approach for Cancer Therapy
Source: PLoS One. 2011 Dec 20;6(12):e28321. doi: 10.1371/journal.pone.0028321 (PMC3243689; doi:10.1371/journal.pone.0028321)
Supplement: Table S1 — Evaluation of apoptosis in hMSCs and SR-4987 after PTX treatment. The evaluation of apoptosis was performed by flow cytometry by the Annexin-V binding assay and confirmed by the quantification of the sub-G1 cell population. Data report the percentage of apoptotic/necrotic cells (mean ± s.d. of three assays). The assays were performed with different passages of SR4987 and different bone marrow donors). CTRL = Untreated cells; PTX 24 h = cells after 24 h of PTX treatment; PTX 24+24 h = treated cells, then subcultured for 24 h without PTX. (DOC) [file pone.0028321.s008.doc]

**Supplementary Table I: Evaluation of apoptosis in hMSCs and SR-4987 after PTX treatment**

| **Cell treatment** | | **Early**  **Apoptosis** | **Late**  **Apoptosis** | **Total**  **apoptosis** | **Necrosis** |
| --- | --- | --- | --- | --- | --- |
| **hMSCs** | *CTRL* | 1.82±0.49 | 7.15±4.53 | 8.97±5.02 | 2.90±1.13 |
|  | *PTX 24h* | 2.60±1.27 | 8.35±1.63 | 10.95±2.90 | 4.60±1.56 |
|  | *PTX 24+24 h* | 5.30±0.28 | 7.90±3.36 | 13.20±3.64 | 5.20±0.42 |
| **SR-4987** | *CTRL* | 5.05±1.13 | 13.40±7.90 | 18.45±5.03 | 6.10±2.20 |
|  | *PTX 24h* | 9.40±4.60 | 21.00±4.10 | ^(*)^30.40±8.70 | 4.50±1.50 |
|  | *PTX 24+24h* | 3.60±0.19 | 13.80±5.87 | 17.40±6.06 | 2.40±0.20 |

^(*)^ p< 0.05 vs CTRL

The evaluation of apoptosis was performed by flow cytometry by the Annexin-V binding assay and confirmed by the quantification of the sub-G1 cell population.

Data report the percentage of apoptotic/necrotic cells (mean ± s.d. of three assays). The assays were performed with different passages of SR4987 and different bone marrow donors).

CTRL= Untreated cells;

PTX 24h= cells after 24h of PTX treatment;

PTX 24+24h= treated cells, then subcultured for 24h without PTX.
