# Supplementary material for: Mesenchymal Stromal Cells Primed with Paclitaxel Provide a New Approach for Cancer Therapy
Source: PLoS One. 2011 Dec 20;6(12):e28321. doi: 10.1371/journal.pone.0028321 (PMC3243689; doi:10.1371/journal.pone.0028321)
Supplement: Text S1 [file pone.0028321.s009.doc]

**Supporting Information**

**mesenchymal sTROMAL PRIMED with paclitaxel provide a new approach for cancer therapy**

Augusto Pessina^1*^, Arianna Bonomi^1^, Valentina Coccè^1^, Gloria Invernici^2^, Stefania Navone ^2^, Loredana Cavicchini^1^, Francesca Sisto^1^, Maura Ferrari^3^, Lucia Viganò^4^, Alberta Locatelli^4^, Emilio Ciusani^5^, Graziella Cappelletti^6^, Daniele Cartelli^6^, Caruso Arnaldo^7^ Eugenio Parati^2^, Roberto Pallini ^8^, Maria Laura Falchetti ^9^ and Giulio Alessandri^2*^

Methods

**Drugs and Reagents**

The stock solution of paclitaxel (PTX) (Serva, Germany) was prepared at the concentration of 5 mg/ml in DMSO and stored at -20°C. Working solutions were freshly prepared according to the experimental design by serial dilutions in culture medium. Fluorescent Paclitaxel Conjugate (PTX-F) (Oregon Green 488 Taxol) and cold PTX for internalization study were purchased from Invitrogen, UK. For MTT assay the Thiazolyl Blue Tetrazolium Bromide (Sigma-Aldrich,Usa) was used.

**Tumor cells**

Human tumor cell lines MOLT-4 (acute lymphoblastic leukemia) [1] were used as reference of the PTX sensitivity and on these cells were normalized all the studies addressed to determine the activity of conditioned media collected from untreated and PTX-primed human mesenchymal stem cells (hMSCsPTX) and mouse stromal cell (SR4987PTX). Other tumor cell lines tested were: T98G (human glioblastoma) [2], DU-145 (human prostate carcinoma) [3] and B16 (mouse melanoma) [4]. All the lines were provided by Centro Substrati Cellulari, ISZLER (Brescia, Italy) and maintained in our laboratory by weekly passages in complete E-MEM supplemented with FCS 10% + 1% Na pyruvate, 1% NEAA (DU-145 and T98G), complete IMDM +10% FCS (MOLT-4), DMEM + 10%FCS (B16) .

HMSCs expansion

HMSCs were prepared from the mononuclear cell fraction of human bone marrow purchased frozen in liquid nitrogen from Lonza (Switzerland) and stored at –120° C until use. The cells, thawed according to a procedure previously described for umbilical cord blood [5] were suspended in Non Hematopoietic Expansion Medium (NHEM, Miltenyi Biotec, Germany), plated in 25 cm^2^ flasks (Corning, USA) at 2 x 10^6^ cells/ml and incubated at 37°C, 5% CO_2_. After 24 hours, floating cells were discarded, medium was replaced and then changed weekly until cells reached 80% confluency. The hMSCs monolayer was trypsinized, subcultured 1:3 and cells expandend until passage number three (PN3) were used in the experiments.

As murine stromal cells was used the line SR4987 established in our laboratory from a long-term bone marrow cells culture [6] and maintained by serial passages in complete RPMI medium + 5% FCS.

Multipotency characterization of hMSCs

HMSCs were tested for their capacity to differentiate into osteocytes, condrocyte and adipocytes according to the methods suggested by [7] with some modifications.

Briefly, cells were plated into 35 mm Petri dishes (Nunc, Germany) at 50 cells/cm^2^ density in 1 ml/Petri of Stemline Mesenchymal Stem Cell Expansion medium (Sigma-Aldrich, St Louis, MO, USA) supplemented with 3% FBS and 4 mM L-Glutamine; after 72 hours of incubation at 37°C, 5% CO_2_, culture medium was replaced with specific differentiation media.

To induce osteogenic differentiation cells were cultured in Stemline supplemented with 10 nM dexamethason, 10 mM glycerol-2-phospate and 300 nM L-ascorbic acid. Differentiation medium was replaced every 3-4 days and after 14 days cultures monolayer were fixed for 5 minutes in cooled methanol at -20°C and then processed for alkaline phosphatase staining, using SIGMA FAST BCIP/NTB (Sigma-Aldrich, USA). To induce chondrocytic differentiation, cells were cultured in hMSC chondrogenesis induction medium (Provitro, Germany) by refeeding cultures every 4 to 5 days. After 24 days, the cultures were fixed for 30 minutes at room temperature with formalin solution, neutral buffered 10% (Sigma-Aldrich, USA) and then processed both by immunocytochemistry technique for aggrecan as follows: cells were permeabilized by 0.3% Triton X-100 for 45 minutes, treated 10 minutes with H_2_O_2_, incubated over/night at 4°C with a mouse anti-human aggrecan monoclonal antibody (1:100, Millipore, USA) and then 1 hour at room temperature with goat anti-mouse HRP-conjugated antibody (1:50, Santa Cruz Biotechnology, CA, USA). Cells were finally developed with 3,3'-Diaminobenzidine (DAB) solution (MP Biomedicals, Canada), 0.5 mg/ml in bidistilled water, for 10 minutes. To induce adipocytic differentiation cells were cultured in Stemline supplemented with indomethacin 200 µM (Alexis Biochemicals, USA), isobutylmethylxanthine 0,5 mM (Applichem, Germany), dexamethasone 1 µM, hydrocortisone 1 µM and insuline 10 µg/ml (all from Sigma-Aldrich, USA). After 10 days of culture, cells were fixed for 30 minutes at room temperature with formalin solution, neutral buffered 10% (Sigma-Aldrich, USA) and then processed for Oil red O staining (Sigma-Aldrich, USA).

# Expression of CD surface markers and P-gp

For characterization of hMSCs, the expression of surface markers was performed by flow cytometry using the following fluorochrome-conjugated mouse anti-human antibodies: CD13-PE, CD14-PE, CD31-PE, CD-33-FITC, CD34-FITC (clone 8G12), CD45-Cy5.5, CD73-PE, CD80-FITC, (Becton Dickinson, USA), CD90-FITC (Miltenyi Biotech, Germany) and CD105-PE (Serotec, Germany).

Cells were harvested and washed once in PBS; 10^6^ cells were incubated, following manufacturer’s instruction, with the antibody at 4°C in the dark for 30 minutes, washed with PBS and analyzed by flow cytometry (FacsVantage SE, Becton Dickinson, USA). For evaluation of P-gp expression, cells were first incubated with a monoclonal antibodies mouse anti-human P-gp (clone C1, Ylem, Italy) for 30 minutes a 4°C, washed with PBS and incubated with a FITC-conjugated goat anti-mouse immunoglobulin (Becton-Dikinson, USA) for further 30 minutes at 4°C. Cell were than washed once and analyzed by flow cytometry. For CD34, CD105 a minimum of 100,000 events per sample were acquired while, for the other markers, 10,000 events per sample were acquired. The analysis was performed using a specific software (CellQuest Pro, Becton Dickinson). Data reported refer to at least two independent experiments.

# Cell cycle analysis and apoptosis

Cells were treated with PTX in 25 cm^2^ flasks according to the above described standard conditions. DNA content for cell cycle phases detection was performed by comparing untreated cells and 24 hours PTX primed cells. Cells were suspended in Phosphate Buffered saline (PBS) and fixed with 96% (v/v) ethanol for 1h at 4°C. After PBS wash, cells were suspended in propidium iodide 50 µg/ml in PBS. Cells were incubated overnight at 4°C and analyzed by flow cytometry (FacsVantageSE, Becton-Dikinson, USA). The evaluation of  apoptosis was performed by flow cytometry by the Annexin-V binding assay (Bender MedSystem, Vienna, Austria) and confirmed by the quantification of the sub-G1 cell population [8] .

**Production of viral stocks and cell infections**

HEK293T, U87MG and SR4987 cell lines were grown in DMEM (high glucose) (Invitrogen Italia, Milano, Italy) supplemented with 10% heat-inactivated FCS. We used a retroviral vector, pRetroQ-DsRed Monomer C1 (Clontech Laboratories, Inc), to infect U87MG cells and a lentiviral vector, pCCLsin.PPT.hPGK.GFPpre [9], to infect SR4987 cells. Amphotropic retrovirus was generated in HEK 293T packaging cells upon cotransfection of the retroviral vector, together with plasmids encoding for gag-pol and VSV-G proteins. Amphotropic lentivirus was generated as described [1]. Briefly, the lentiviral construct was cotransfected in HEK 293T with pMDL, pRSV-REV and pVSV-G. Viral supernatants were collected 48 h post-transfection, filtered through a 0.45 μm pore size filter and added to U87MG and SR4987 cells in the presence of 2 μg/ml and 8 μg/ml polybrene respectively. Cells were subjected to three consecutive rounds of infection. Puromycin resistance was used to select U87MG infected cells. Lentiviral infection occurred with high efficiency, as assessed by GFP fluorescence, so that no enrichment for transduced cells was required.

PTX internalization

PTX internalization was initially evaluated by confocal microscopy. hMSCs were plated onto poly-L-lysine-coated glass bottom dishes (MatTek, Ashland, MA) and primed with PTXF in standard conditions as above described. Immediately before the observation, cells were loaded for 15 minutes with 4 μM BOPIPY TR ceramide (Invitrogen, UK) to stain Golgi apparatus and Golgi derived vesicles. Cells were washed two times in PBS and once in culture medium, and then living cells were observed with a confocal laser scan microscope (TCS SP2 AOBS, Leica), by using a 63X immersion oil objective.

The internalization of PTX by the cells was confirmed and the kinetics of PTX decrease was shown by FACS analysis priming hMSCs with unlabelled PTX (controls) and PTXF in standard conditions as above described. Cells were than harvested, washed three times with PBS, resuspended in complete medium and kept in suspension at 37°C in 5% CO_2_. Residual green fluorescence was detected by flow cytometry (FacsVantage SE, Becton Dickinson, USA), immediately after treatment and after 2, 4, 8, 24 and 48 hours. Data were expressed as ratio between mean fluorescence intensity of PTXF-treated hMSCs and mean fluorescence intensity of PTX-treated hMSCs at the various time-points.

Procedure for PTX priming and testing drug release by hMSCs and SR4987

10^5^ hMSCs and 10^5^ SR4987 were seeded in 25 cm^2^ flask in 5 ml of NHEM medium. After 72-96 hours, to the medium was added PTX at a final concentration of 2.000 ng/ml and cells were cultured for further 24 hours at 37 °C in atmosphere of air + CO_2_ 5%. At the end of the incubation, the cell supernatant was discarded, the cell monolayer was washed twice with PBS and then trypsinized (0.025 % trypsin + 0.01 % EDTA) for 5 minutes. When 80% of the cells were detached a complete DMEM medium + 20% FCS was added, the cells were collected by centrifugation, washed twice in HBSS and after counting were seeded into a 25 cm^2^ flask. After 24 hours of culture the cell conditioned medium (CM) was collected and replaced by repeating this procedure at 48, 72, 96 and 144 hours. The CM collected as described were then tested for their anti-proliferative activity *in vitro* on the different tumor cell lines. As negative controls were used CM from hMSCs and SR4987 processed as above described but without receiving PTX.

The following different experimental conditions were verified:

1. Passive membrane drug adsorption and release: to verify the amount of drug passively adsorbed and then eluted from the cell membrane, hMSCs and SR4987 monolayer was fixed with formalin solution, neutral buffered, 10% (Sigma-Aldrich, Usa) for 30 minutes, washed three time with bidistilled water and then treated with PTX as above described.
2. Drug upatke and release in suspension: hMSCs and SR4987 were maintained stirred into 15 ml conic tube to prevent their adhesion and verify if the PTX-uptake during the drug treatment was dependent on the cell adhesion. In opposite protocol the hMSCs and SR4987 treated with PTX, as described in the main procedure, were trypsinized and then maintained stirred for 24 hours to prevent substrate adhesion of the cells. The cells were then centrifuged and the supernatant tested to verify if PTX-release by cells was dependent on their adhesion.

*In vitro* anti proliferative assay on tumor cell lines of PTX and CM from PTX primed hMSCs and SR4987

The effect of both PTX and Conditioned Medium from PTX- treated hMSC (hMSCsPTX-CM) and from PTX-treated SR4987 (SR4987PTX-CM) on tumor cell proliferation has been studied in 96 multiwell plates (Sarstedt, Germany) by using as target cells MOLT-4, T98G and DU-145 cells. Briefly, 1:2 serial dilutions of drugs or hMSC-CM or SR4987-CM were prepared in 100 µl of culture medium/well and then to each well were added 1,000 tumor cells. After 7 days of culture at 37°C, 5% CO_2_ cell viability was evaluated by the MTT (3-(4,5-dimethyl-2-thiazolyl)-2,5-diphenyl-2-H-tetrazolium) assay as previously described [10,11]. For each cell line and drug the inhibitory concentrations (IC) 50 and 90 were determined according to the Reed and Muench formula [12] and also by using the linear regression analysis on the dose-response kinetics of the inhibition .

The antitumoral activity of hMSCsPTX-CM and SR4987PTX-CM were compared to the one of pure PTX and expressed as PTX equivalent concentration (PEC) according to the following algorithm

PEC(ng/ml) = DF_50_CM x IC_50_PTX

where the DF_50_CM is the dilution factors (DF) at which the 50% of inhibition was observed with CM from primed hMSCs or SR4987 cells and IC_50_ is the concentration (ng/ml) of pure PTX producing 50% of inhibition. To evaluate the single cell PTX release (PR) by primed MSCs or SR4987 cells we used an arbitrary internal parameter based on the ratio between the PEC and the number of cells seeded, according the following formula: PR (pg/ml) = PEC (ng/ml) x CM volume (ml) / number of cell seeded.

*In vitro* inhibition of tumor growth by PTX primed hMSC or SR4987

To verify the ability of hMSCs and SR4987 to inhibit the i*n vitro* tumor cell proliferation a co-culture system was applied by mixing 1,000 tumor cells with different amount of cells (1,000-100 or 10 cells) in order to have a final proportion tumor cells/hMSCs (tumor cells/SR4987) of 1-10-100. Three experiments were conducted in duplicate in a volume of 100 μl/ well: one receiving tumor cells and untreated hMSCs (or untreated SR4987) (control), one by mixing tumor cells with hMSCs treated with PTX (MSCsPTX) or with SR4987 treated with PTX (SR4987PTX) as above described. Further control wells received 100 μl of hMSCs or SR4987 alone at the three different amounts or only 1,000 cells /well of tumor cells. The cultures were incubated 7 days in air CO_2_ at 37 °C and then the cell growth evaluated in a MTT assay as above described. The results were expressed as percentage of the proliferation observed in tumor cell culture that did not receive hMSCs or SR4987 cells (considered as 100%). Arbitrary values of IC_50_ and IC_90_ were calculated as ratio hMSCs/tumor cells (SR4987/tumor cells) able to inhibit respectively the 50 and the 90 % of tumor cell proliferation.

PTX dosage by HPLC

PTX was measured in hMSCsPTX-CM or SR4987PTX-CM by a validated high performance liquid chromatography (HPLC) method over the concentration range of 12.5 to 1.000 ng/mL.

After addition of cephalomannine (Sigma, St Louis, MO) as internal standard (final concentration 200 ng/mL), 5 mL of CM was extracted on a Bond Elut LRC C-18 solid-phase extraction cartridge (Varian- Harbor City, CA) and, after evaporation to dryness, the samples were reconstituted in 100 µL of mobile phase and analyzed on the chromatographic system (LC-1200, Agilent Technologies, Santa Clara, CA). Chromatographic separation was achieved on a Zorbax RX- C18 analytical column (Agilent Technologies) using a mobile phase containing 48% acetonitrile–52% distilled water. Detection was by UV absorbance at 230 nm. The standard curve was fitted to a weighted (1/Y^2^) linear regression curve [13].

Evaluation of anti-angiogenic properties of SR4987 and hMSCs primed with PTX *in vitro*

To assess the anti-angiogenic potential of SR4987 and hMSCs primed or not with PTX, initially we tested the effect of PTX and CM (collected after 24 hours of culture of both control and PTX treated MSCs) on the proliferation of Human Umbilical vein ECs (HUVEC) (purchased from Lonza) and on human derma microvascular ECs (HMECs) isolated as previously described [14]. Both ECs phenotypes were routinely maintained in EGM bullet kit plus 10% FCS (Lonza). ECs proliferation assay was performed as described [12]. Briefly HUVECs and HMECs (at passage 3) were harvested from culture flask by trypsin. After enzyme inactivation and centrifugation, cells were resuspended in EGM medium + 0.2% BSA and counted. To evaluate the growth response to control SR4987-CM, hMSCs-CM and primed SR4987PTX-CM, hMSCsPTX-CM, 0.5 ml of HUVECs and HMECs (10^4^ cells) were seeded into each well of a 24-multiwell plate coated with collagen type I; after cell adhesion, medium was aspirated and replaced with EGM complete medium supplemented or not with different dilution (ranging from 1:2 to 1:8) of control SR4987-CM, hMSCs-CM and primed SR4987PTX-CM and hMSCsPTX-CM. Negative control consisted in the addition of PTX (from 1 to 100 ng/ml), while positive control growth medium consisted in EGM + 5ng/ml of VEGF-A (Lonza). After 72 h, the wells were washed, fixed and stained. The cells were counted with a calibrated ocular eyepiece in 10 different fields at 400X magnification. The capacity of SR4987PTX and hMSCsPTX to inhibit ECs proliferation was also tested by co-culture assay. As described above for tumor cells, ECs were mixed with control SR4987 and hMSCs and primed SR4987PTX and hMSCsPTX at different ratio, ranging from 1:1 to 1:10 MSCs/ECs. Inhibition of HUVECs and HMECs proliferation was estimated by counting after immunofluorescent staining the CD31 [15] positive cells in the culture in the presence of control hMSCs and hMSCsPTX. Negative control consisted in the addition of 5 ng/ml of PTX to EC culture. Pictures of the co-culture were taken at different time of incubation .

The rat aorta ring assay was also performed to investigate the anti-angiogenic potential of control and hMSCsPTX-CM. The aorta ring assay was performed as previously described (16). Briefly the dorsal aorta was excised from 6 weeks old Sprague-Dawley rats. Around 1 mm-thick rings were prepared. The rings were placed in collagen gel prepared as described [17]. Around 40 μl of collagen solution were placed in each well of a 4 multiwell plates and than one aorta ring/well was placed into the collagen solution. The plates were than incubated in humidified incubator at 37°C for 30’ to obtain collagen jellification. At the end of incubation the wells were filled with 500 μl of EBM (LONZA) medium containing different dilution of hMSCs-CM, hMSCsPTX-CM and 20 ng/ml of VEGF were used as positive control. The plates were incubated for 10-12 days. Quantification of angiogenesis was obtained by taking photographs every three days and by counting the number of microvessels arising from aorta rings [16].

Evaluation of anti-tumor activity of hMSCsPTX and mouse SR4987PTX *in vivo*

The capacity of control hMSCs and hMSCsPTX to affect DU145 and B16 melanoma growth were performed on 8 weeks old male NOD/SCID mice (JAX Mice: NOD.CB17-Prkdc scid/j e) and Nude mice (4-week-old male athymic nude-Foxn1 nu/ nu from Harlan Italy) respectively. Eight weeks old male C57Bl6 (Charles River, Italy) were used to evaluate the effect of mouse control SR4987 and SR4987PTX cells on B16 melanoma. Control hMSCs and hMSCsPTX were detached with trypsin from culture flask and centrifuged. Each cell pellet was resuspended in PBS and mixed with DU145 and B16 melanoma to obtain the final ratio MSCs/tumor cells 1:5 consisting of injecting in the subcute of each mouse 0,4 x 10^6^ control hMSCs and primed hMSCsPTX with 2 x 10^6^ DU145 or with B16 melanoma in a total volume of 0.2 ml. Similar procedure for SR4987 and SR4987PTX that were mixed at ratio 1:5 with B16 melanoma and injected in the subcute of singenic C57BL/6. In this case each mouse received 0.4 x 10^5^ control and SR4987PTX and 2 x 10^5^ B16 cells. Control mice groups received either DU145 and B16 melanoma alone or DU145 and B16 mixed with a solution of 2 μg/ml of PTX before injection. After cells injection of the flanks of animals, they were observed every day to establish tumor appearance, thereafter tumor size were measured every 2-3 days with a callipers and volume was calculated by the formula D x d^2^ / 2, where D and d are the major and the minor diameter of tumor respectively. At the end of experiments mice were sacrificed and subacute tumour removed and weighted. All transgenic animals were purchased from the Jackson Laboratory (Bar Harbor, ME). All animal experiments were performed according to institutional guidelines that are in compliance with national (D.I. no. 116, G.U. suppl. 40, Feb. 18, 1992, Circolare No.8, G.U., 14 Luglio 1994) and international law and policies (EEC Council Directive 86/609, OJ L358, 1 Dec. 12, 1987; Guide for the Care and Use of Laboratory Animals, U.S. National Research Council, 1996).

**Subcutaneous grafting of RFP+ U87MG glioblastoma cells and GFP+ SR4987 cells primed with PTX**

Experiments involving animals were approved by the Ethical Committee of the Catholic University School of Medicine, Rome. Nude athymic mice (4–6 weeks of age; HDS-athymic nude mice, Charles Rives, Milan, Italy) were implanted subcutaneously on both sides with either 1 x 10^6^ RFP+ U87MG cells (*n*, 2), or with 1 x 10^6^ RFP+ U87MG cells and 2 x 10^5^ GFP+ SR4987 (*n*, 8), or with 1 x 10^6^ RFP+ U87MG cells and 2 x 10^5^ GFP+ SR4987 cells primed with PTX( GFP+SR4987PTX) (*n*, 6). Control mice were grafted either with 1 x 10^6^ U87MG cells, or with 1 x 10^6^ U87MG cells and 2 x 10^5^ SR4987. For grafting, the cells were resuspended in 0.1 ml of cold phosphate buffered saline and the suspension was mixed with an equal volume of cold Matrigel (BD Bioscience, Bedford, MA). Mice were kept under pathogen-free conditions in positive-pressure cabinets (Tecniplast Gazzada, Varese, Italy) and observed daily for the visual appearance of tumors at injection sites. Tumor diameter was measured using callipers and calculated as the mean value between the shortest and the longest diameters. By 2, 4, and 6 weeks after grafting, the mice were deeply anesthetized and transcardially perfused with 0.1 M PBS (pH 7.4), followed by 4% paraformaldehyde in 0.1 M PBS. The subcutaneous xenografts were removed, stored in 30% sucrose buffer overnight at 4°C, and serially cryotomed at 20 microm. Sections were collected in distilled water, mounted on slides, and cover-slipped with Eukitt. Images were obtained with a Laser Scanning Confocal Microscope (IX81, Olympus Inc, Melville, NY). Alternate sections were stained with H&E. To assess the mesenchymal phenotype of engrafted GFP+SR4987, tumor sections were immunostained with mouse monoclonal anti-vimentin antibody (Dako, Glostrup, Denmark) using the Vector VIP substrate kit for peroxidase (Vector Laboratories, Inc., Burlingame, CA).

References

1. Minowada J, Onuma T, Moore GE (1972) Rosette-forming human lymphoid cell lines. I. Establishment and evidence for origin of thymus-derived lymphocytes. J Natl Cancer Inst 49: 891-895.

2. Stein G (1979) T98G: an anchorage-independent human tumor cell line that exhibits stationary phase G1 arrest in vitro. J Cell Physiol 99: 43-54.

3. Mickey DD, Stone KR, Wunderli H, Mickey GH, Vollmer RT et al. (1977). Heterotransplantation of a human prostatic adenocarcinoma cell line in nude mice. Cancer Res 37: 4049-4058.

4. Riley V (1963) Enzymatic determination of transmessible replicating factors associated with mouse tumors. Ann N Y Acad Sci 100: 762-790.

5. Pessina A, Eletti B, Croera C, Savalli N, Diodovich C et al. (2004) [Pancreas developing markers expressed on human mononucleated umbilical cord blood cells.](http://www.ncbi.nlm.nih.gov/pubmed/15351739) Biochem Biophys Res Commun 323: 315-322.

6. Pessina A, Mineo E, Neri MG, Gribaldo L, Colombi R et al. (1992) [Establishment and characterization of a new murine cell line (SR-4987) derived from marrow stromal cells.](http://www.ncbi.nlm.nih.gov/pubmed/1382506) Cytotechnology 8: 93-102.

7. [Pittenger MF](http://www.ncbi.nlm.nih.gov/pubmed?term="Pittenger MF"%5BAuthor%5D), Mackay AM, Beck SC, Jaiswal RK, Douglas R et al. (1999) Multilineage potential of adult human mesenchymal stem cells. [Science](javascript:AL_get(this, 'jour', 'Science.');) 284: 143-147.

8. Shumilina E, Thi Xuan N, Schmid E, Bhavsar SK, Szteyn K et al. (2010) Zinc induced apoptotic death of mouse dendritic cells. Apoptosis 15:1177–1186.

9. Dull T, Zufferey R, Kelly M, Mandel RJ, Nguien M et al. (1998) A third-generation lentivirus vector with a conditional packaging system. J Virol 72:8463-71.

10. Mossman T (1983) Rapid colorimetric assay for cellular growth and survival: application to proliferation and cytotoxicity assays. J Immunol Methods 65: 55-63.

11. Pessina A, Gribaldo L, Mineo E, Neri MG (1994) In vitro short-term and long-term cytotoxicity of fluoroquinolones on murine cell lines. Indian J Exp Biol 32: 113-118.

12. Reed LJ, Muench H (1938) A simple method of estimating fifty percent endpoints. The American Journal of Hygiene 27: 493-497.

13. Gianni L, **Kearns CM, Giani A, Capri G, Vigano L** et al. (1995) Nonlinear pharmacokinetic of paclitaxel and its pharmacokinetic/ pharmacodynamic relationships in humans. J Clin Oncol 13: 180-190.

14. Caruso A, **Rotola A, Comar M, Favilli F, Galvan M** et al. (2002) HHV-6 infects human aortic and heart microvascular endothelial cells increasing their ability to secrete proinflammatory chemokines. J Med Virol 67: 528-533.

15. Invernici G, **Emanueli C, Madeddu P, Cristini S, Gadau S** et al. (2007) Human fetal aorta contains vascular progenitor cells capable of inducing vasculogenesis, angiogenesis, and myogenesis in vitro and in a murine model of peripheral ischemia. Am J Pathol 170: 1879-1892.

16. Nicosia RF, Ottinetti A. (1990) Growth of microvessel in serum-free matrix culture of rat aorta: a quantitative assay of angiogenesis in vitro. Lab Invest 63:115-122.

17. Elsdale T, Bard J (1992) Collagen substrata for study of cell behavior. J Cell Bio 54: 5539-555l.
